# Improving landslide susceptibility prediction through ensemble recursive feature elimination and meta-learning framework

**DOI:** 10.1038/s41598-025-87587-3

**Published:** 2025-02-12

**Authors:** Krishnagopal Halder, Amit Kumar Srivastava, Anitabha Ghosh, Subhabrata Das, Santanu Banerjee, Subodh Chandra Pal, Uday Chatterjee, Dipak Bisai, Frank Ewert, Thomas Gaiser

**Affiliations:** 1 Department of Remote Sensing and GIS, Vidyasagar University, Vidyasagar University Rd, Midnapore, 721102 West Bengal India; 2 Institute of Crop Science and Resource Conservation, University of Bonn, Katzenburgweg, 5D-53115 Bonn, Germany; 3 Langmuir Center of Colloids and Interfaces, Columbia University in the City of New York, New York, 10027 USA; 4 College of Agriculture, Chhatrapati Shahu Ji Maharaj University, Kanpur, 208012 UP India; 5 Department of Geography, The University of Burdwan, Barddhamān, India; 6 Department of Geography, Bhatter College, Dantan, Kharagpur, 721426 West Bengal India; 7 Coastal Environmental Studies Research Centre, Egra S.S.B. College, (Affiliated to Vidyasagar University), Kharagpur, 721429 West Bengal India; 8 Leibniz Centre for Agricultural Landscape Research (ZALF), Eberswalder Strasse 84, 15374 Müncheberg, Germany

**Keywords:** Landslide, Machine learning, Recursive feature elimination, meta-learning framework, Environmental impact, Natural hazards

## Abstract

Landslides pose significant threats to ecosystems, lives, and economies, particularly in the geologically fragile Sub-Himalayan region of West Bengal, India. This study enhances landslide susceptibility prediction by developing an ensemble framework integrating Recursive Feature Elimination (RFE) with meta-learning techniques. Seven advanced machine learning models- Logistic Regression (LR), Support Vector Machine (SVM), Random Forest (RF), Extremely Randomized Trees (ET), Gradient Boosting (GB), Extreme Gradient Boosting (XGBoost), and a Meta Classifier (MC) were applied using Remote Sensing and GIS tools to identify key landslide-conditioning factors and classify susceptibility zones. Model performance was assessed through metrics such as accuracy, precision, recall, F1 score, and AUC of the ROC curve. Among the models, the Meta Classifier (MC) achieved the highest accuracy (0.956) and AUC (0.987), demonstrating superior predictive ability. Gradient Boosting (GB), XGBoost, and RF also performed well, with accuracies of 0.943 and AUC values of 0.987 (GB and XGBoost) and 0.983 (RF). Extremely Randomized Trees (ET) exhibited the highest accuracy (0.946) among individual models and an AUC of 0.985. SVM and LR, while slightly less accurate (0.941 and 0.860, respectively), provided valuable insights, with SVM achieving an AUC of 0.972 and LR achieving 0.935. The models effectively delineated landslide susceptibility into five zones (very low, low, moderate, high, and very high), with high and very high susceptibility zones concentrated in Darjeeling and Kalimpong subdivisions. These zones are influenced by intense rainfall, unstable geological structures, and anthropogenic activities like deforestation and urbanization. Notably, ET, RF, GB, and XGBoost demonstrated efficiency in feature selection, requiring fewer input variables while maintaining high performance. This study establishes a benchmark for landslide susceptibility mapping, providing a scalable and adaptable framework for geospatial hazard prediction. The findings hold significant implications for land-use planning, disaster management, and environmental conservation in vulnerable regions worldwide.

**Supplementary Information:**

The online version contains supplementary material available at 10.1038/s41598-025-87587-3.

## Introduction

A landslide is a geological occurrence in which a large amount of rock, soil, or debris descends a slope, due to the force of gravity, and may arise as a consequence of substantial precipitation^1^. Landslides have a profound effect on people’s lives by demolishing residences and assets, interrupting transportation and trade, resulting in economic loss, reducing biodiversity and ecological functions, and impairing public services. Landslides pose a significant threat to a vast expanse of the land surface, that extends 3.8 million Km^2^^2^. This poses a risk to the lives and livelihoods of approximately 300 million individuals, accounting for roughly 6% of the global population. The soil thematic strategy of the European Union (EU) acknowledges landslides as one of the eight types of soil erosion concerns and promotes the identification of landslide-prone areas^3^. Landslides and avalanches are significant hydro-geological hazards that impact extensive areas of India, including the Himalayas, the Northeastern hill ranges, the Western Ghats, the Nilgiris, the Eastern Ghats, and the Vindhyans^4^. While it is impossible to prevent natural hazards like landslides, understanding trends, and scientific findings can help us anticipate and reduce our vulnerability to similar events.

Landslide susceptibility models may be developed using several approaches, including multi-criteria decision-making methods based on expert opinions, statistical methods, and machine learning models. Several commonly employed methods for multi-criteria decision-making and statistical methods include Multi-Criteria Decision-Making (MCDM)^5–8^, Analytical Hierarchy Process (AHP)^9^, Evidential Belief Function (EBF)^10^, Shannon Entropy (SE)^1,11^. However, Machine Learning (ML) and Deep Learning (DL) algorithms have been greatly employed in recent years to construct landslide susceptibility models^12^. These methods build models based on the assumption that the conditions leading to landslides have causal relationships with historical events. Diverse algorithms have effectively been used in the investigation of creating landslide susceptibility maps: Adaptive Boosting (AdaBoost)^13,14^, Artificial Neural Network (ANN)^15,12^, Convolutional Neural Network (CNN)^16,17^, Deep Neural Network (DNN)^18,17^, MultiLayer Perceptron Neural Network (MLP)^19,20^, however, the utilization of a meta classifier, instead of relying solely on individual models, always gives a strategic advantage in improving the predictive performance and robustness of complex machine learning tasks. Although individual models demonstrate proficiency in capturing some features of the data, their shortcomings become evident when confronted with different patterns or noisy elements in the dataset. For example, linear models reveal limitations when dealing with non-linear datasets. Support vector machines (SVM) may have some challenges in terms of computing complexity, particularly with large datasets, and can be susceptible to noisy input. Therefore, recent advancements in landslide prediction modeling have shown promise with hybrid machine learning algorithms, as indicated by^60^. The utilization of a hybrid technique, as demonstrated by^38^ enhances the prediction performance of the base model.

In the present study, six advanced machine learning (ML) models including Logistic Regression (LR)^16,21^, Support Vector Machine (SVM)^22^, Random Forest (RF) [15, 61), Extremely Randomized Trees (ExtraTrees)^23,14^, Gradient Boosting (GB)^24,25^, Extreme Gradient Boosting (XGBoost)^26,19,27,28^ and a Meta Classifier (MC), which integrates the prediction of six distinct models, were adopted to construct landslides susceptibility maps using advanced Remote Sensing (RS) and Geographic Information System (GIS) techniques. The implementation of an ensemble feature selection process in our study reflects a pioneering approach to landslide susceptibility mapping. To our knowledge, this approach has not been explored within this domain. This new feature selection technique strengthens the robustness of our model by collectively finding and including the most significant causative aspects of landslides. Subsequently, we constructed a meta-model by stacking predictions from the six models, resulting in more trustworthy landslide susceptibility maps. This constitutes a novel and distinctive component of our research, offering a fresh viewpoint on enhancing the accuracy and reliability of landslide susceptibility mapping.

### Study area

**Fig. 1:**
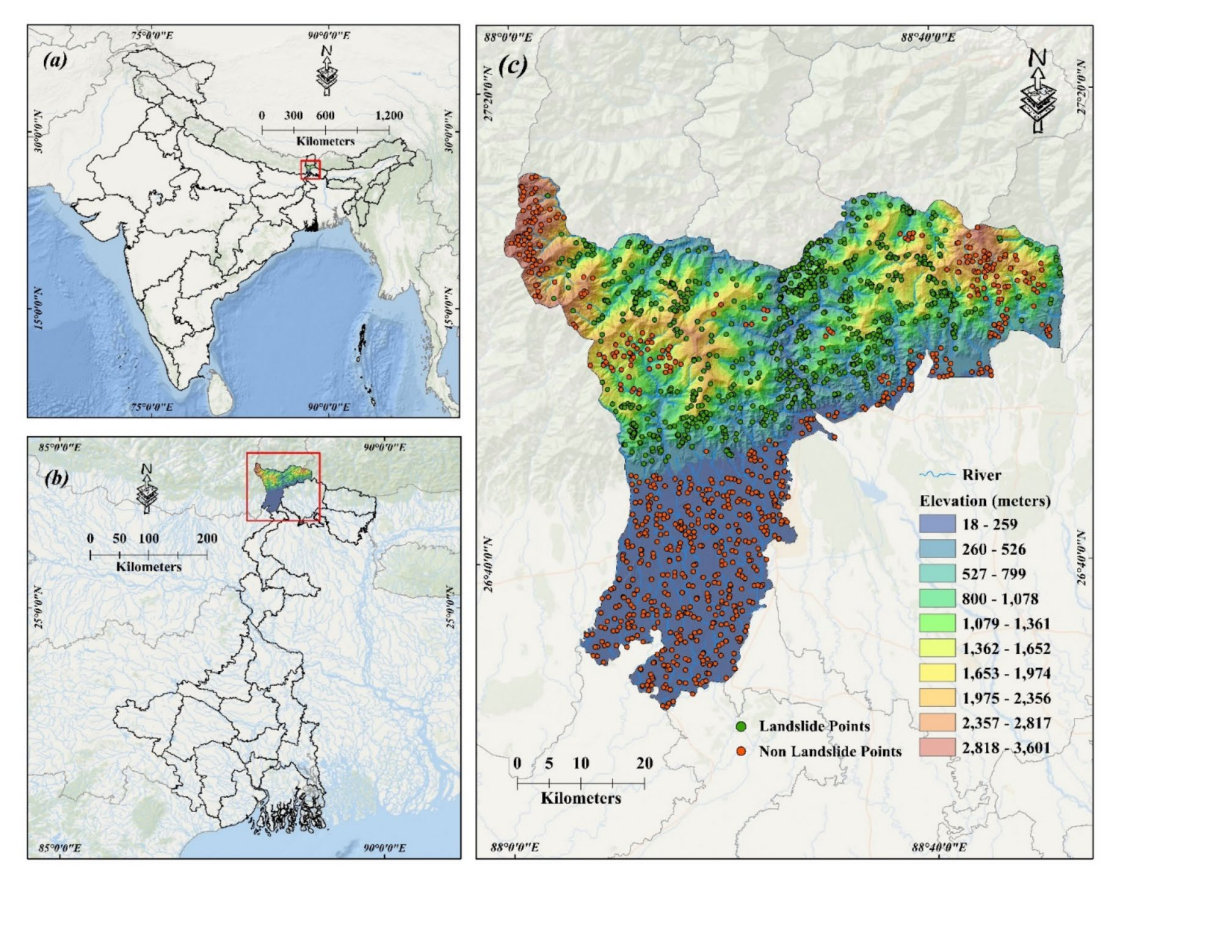
Location map of (**a**) India, (**b**) West Bengal, and (**c**) Study Area. [Created by ArcGIS 10.4.1; Esri. (2016). ArcGIS Desktop: Release 10.4.1 [Software]. Environmental Systems Research Institute. https://www.esri.com ].

**Fig. 2:**
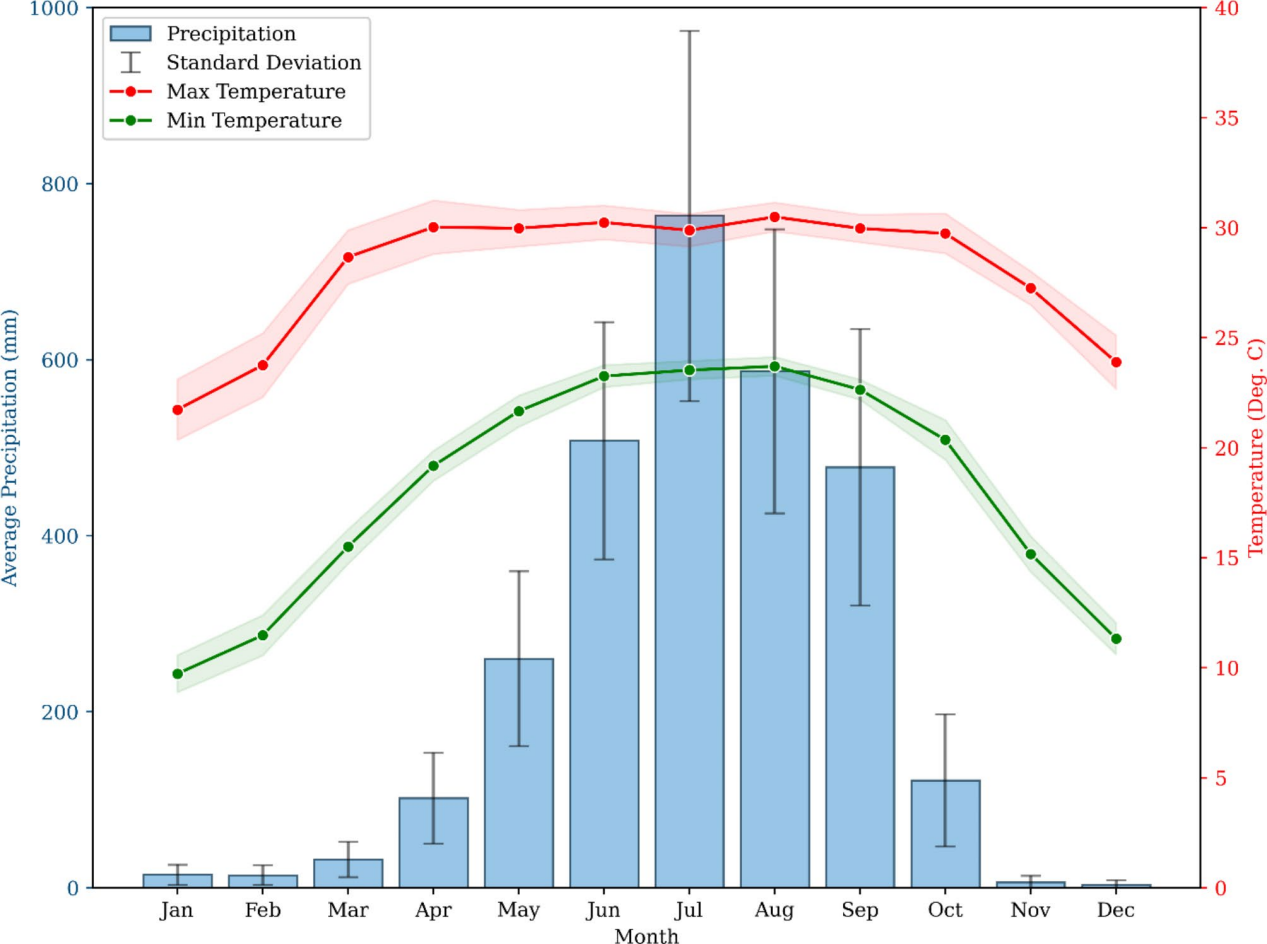
Average precipitation (mm), maximum and minimum temperature (°C) over 32 years (1991–2022).

The study area is situated in the northernmost region of West Bengal, India (Fig. 1a,b). The Darjeeling and Kalimpong districts are located in the eastern Himalayas area of India and are mostly characterized by steep and harsh mountainous topography. The study area is extended between the latitudes 26°27″ to 27°13″N and longitudes 87°59″E − 88°53″E. The study area spans a total of 3149 square kilometers (Fig. 1c) and exhibits an altitude range of 15 m to 3616 m above mean sea level, with slopes ranging from about 0° to 80°. The climate in the hill area is warm and humid, with moderate summers (Köppen climate classification: Cwb) when the maximum temperature rarely exceeds 25 °C. In contrast, the lowlands endure hot and humid conditions, with maximum temperatures reaching as high as 42 °C. The monsoon season, which occurs from June to September, is distinguished by heavy monsoon rains. The research region has an average yearly precipitation of around 3051 mm (Fig. 2). The Darjeeling hills have been formed by an accumulation of folds, faults, and tangential thrusts resulting from compression in the north-south direction, as the Indian tectonic plate subducts under the Eurasian plate. The cumulative geological processes have subjected the rocks to shearing, folding, compression, fracturing, and jointing, resulting in a decrease in their structural integrity. The occurrence of landslides posed a significant and concerning issue in the region. An abundance of rainfall, seismic activity, and rapid erosion could be the potential triggering factors for the initiation of many of these events. The acceleration of these processes is caused by widespread deforestation, inadequate drainage systems, substandard revetments, and the existence of steep slopes that have been excavated to create platforms for pathways, roads, and buildings.

### Landslide inventory map

An accurate depiction of the region’s past and present landslide events is essential for evaluating the possible occurrence of landslides and ascertaining their susceptibility. The creation of a map depicting the location, type, magnitude, and date of occurrence of landslides involves the use of field surveys, remote sensing technology, and historical analysis. Hence, it is crucial to develop a comprehensive and precise landslide inventory map to assess landslide susceptibility and examine the correlation between landslide occurrences and their triggering factors. The landslide inventory map created using historical data from BHKOSH-GSI (Geo-platform of the Indian Space Research Organization) was combined with the study area map (Fig. 1c). Non-landslide points were generated by creating a 2 km buffer around each landslide location and then randomly generating points outside of these buffer zones. This method ensures the generation of non-landslide locations in areas that are different from the vicinity of landslide occurrences. This helps in creating a balanced and representative dataset for conducting a thorough binary classification task. To train and validate the models, the whole dataset was randomly partitioned into two parts: Training data covers 70% (1020 landslide points), and validation data covers 30% (462 landslide points) of the whole dataset, respectively^29,30^. In machine learning and spatial modeling research, the 70/30 split is a commonly used approach because it maintains the ideal balance between having enough data to train the model and keeping enough unseen data for reliable model validation.

## Methodology

### Data acquisition and preparation of thematic maps

Landslide occurrences result from complex interactions among various topographical, hydrological, climatic, geological, land cover, and anthropogenic factors. Mapping landslide susceptibility involves considering multiple factors, each with distinct impacts, necessitating a thorough review of literature, expert consultations, and on-site assessments to identify suitable criteria. Prior research has outlined key aspects influencing landslide susceptibility mapping, informing the parameters utilized in susceptibility models. Over the past two decades, geospatial methodologies have been extensively employed to analyze risks, vulnerabilities, and hazards efficiently.

In this study, thematic layers were created in ArcGIS 10.4.1 by using a geographical database including 21 parameters that contribute to landslide susceptibility conditions. The ASTER GDEMs (Advanced Spaceborne Thermal Emission and Reflection Radiometer Global Digital Elevation Model) were merged using the “Mosaic to New Raster” tool in the “Data Management Tools” and then adjusted using the “Hydrology” tool in the “Spatial Analyst Tools” to clip and fill them. Topographical factors like elevation, slope, slope aspect, profile curvature, roughness, TPI, and TRI were derived from ASTER GDEM (30 m x 30 m). Hydrological factors like drainage density, distance to river, SPI, STI, and TWI were derived from ASTER GDEM (30 m x 30 m). Landcover factors like NDVI, MNDWI, and LULC were radiometrically corrected and calculated in “Raster Calculator” by using Landsat-8 OLI/TIRS, (30 m x 30 m) satellite images. Distance to Road derived from OpenStreetMap (OSM), then distance to the road map created using the “Euclidean distance” function in the “Spatial Analyst Tool.” Annual Rainfall was prepared using gridded rainfall (0.25° x 0.25°) available from the Indian Meteorological Department (IMD) (Table 1). To determine the extent of the effect of a rain gauge station, Thiessen polygons were first generated using the “Proximity” function in the “Analysis Tools” of ArcGIS 10.4.1. The soil Texture map was derived from the digital map of the district, sourced from the National Bureau of Soil Survey and Land Use Planning (NBSSLUP). Geological factors like lithology, geomorphology, and lineament density were derived from a digital map of the district, provided by the Geological Survey of India (GSI). Finally, all layers were resampled into the “Reclassify” function in the “Spatial Analyst Tools” in ArcGIS 10.4.1 into 30-m spatial resolution and further incorporated according to the methodology of the Machine Learning (ML) algorithms to finalize the landslide susceptibility zonation of the region.

**Table 1:** Source and description of the parameters used in landslide susceptibility.

Parameters	Descriptions	Source
Elevation, slope, slope aspect, roughness, SPI, STI, TPI, TRI, TWI, drainage density and distance to river	Derived from ASTER DEM (30 m x 30 m) and prepared the thematic layer using ArcGIS 10.4.1	United States of Geological Survey (USGS) Retrieved from: https://earthexplorer.usgs.gov
NDVI, mNDWI and LULC	Landsat-8 OLI/TIRS, (30 m x 30 m)	United States of Geological Survey (USGS) Retrieved from: https://earthexplorer.usgs.gov
Rainfall	Gridded rainfall (0.25° x 0.25°) NetCDF File	Indian Meteorological Department (IMD) Retrieved from: http://www.imdpune.gov.in
Geomorphology, lithology, and lineament	Digital map of the district	Geological Survey of India (GSI) Retrieved from: http://bhukosh.gsi.gov.in/
Soil texture	Digital map of the district	National Bureau of Soil Survey and Land Use Planning Retrieved from: http://www.nbsslup.in
Distance to road	Adopting the data from OpenStreetMap	OpenStreetMap Retrieved from: www.openstreetmap.org

### Preprocessing of explanatory variables

In the context of enhancing the efficacy of landslide susceptibility prediction, we deliberately selected 21 (refer to Supplementary Figure S1) landslide conditioning factors (LCFs) that reflect the unique characteristics of the geographic area under investigation. The landslide conditioning factors (LCFs) investigated in this research have a significant influence on landslide calamities^15,16^. Consequently, it is necessary to analyze the correlation between the results and the distribution of factors. While the specific function of LCFs may differ depending on the area, it is undeniable that a combination of geo-environmental factors serves as a regulator for landslides^31^. Choosing the appropriate landslide conditioning factors (LCFs) in landslide hazard modeling enhances the accuracy of the findings and reduces interference, hence enhancing the predictive capabilities of the model^31^. Nevertheless, it is widely acknowledged that there is no established protocol or criterion for choosing LCFs. Twenty-one LCFs were selected as an independent landslide conditioning factor (LCFs) to assess the susceptibility to landslides in the Sub-Himalayan region of West Bengal, India. Once the most important landslide conditioning factors (LCFs) were identified, the dataset was divided into a training set (70%) and a testing set (30%) for the 1082 locations of landslides and non-landslides. The division was done randomly. Empirical research consistently demonstrates that allocating 20–30% of the data for testing and the remaining 70–80% for training yields optimal outcomes^24,16,19,22,14^. In order to address the problem of underfitting and overfitting caused by the dataset size, this work conducted a series of experiments with different proportions of training and testing data (70:30, 75:25, and 80:20). During this inquiry, it was determined that a ratio of 70:30 was deemed appropriate. The precision achieved with a 70:30 split produces superior outcomes compared to the 80:20 and 75:25 divisions.

Feature scaling is an important data preprocessing step that ensures the equitable contribution of each feature to the learning process. Many machine learning algorithms use distance-based metrics, such as Euclidean distance, to classify different features. Therefore, the magnitude of features significantly affects their influence on model training. Models may exhibit biased and inferior performance when features of larger scales overpower those of lower sizes.

As seen in Eq. 1, normalization, a popular feature scaling technique, was employed to normalize explanatory variables to a similar range (0 to 1), facilitating optimization convergence and improving model interpretability:

1 $$\:{X}_{normalized}=\frac{X-{X}_{min}}{{X}_{max}-{X}_{min}}$$

Nevertheless, not every machine-learning model demands feature scaling. For instance, algorithms like logistic regression, support vector machines (SVM), multilayer perceptrons (MLP), and k-nearest neighbors (kNN) exhibit better performance when feature scaling is implemented. In contrast, tree-based models like decision trees, random forests, and gradient boosting are not affected by feature scaling and maintain their performance irrespective of this preprocessing step.

**Fig. 3:**
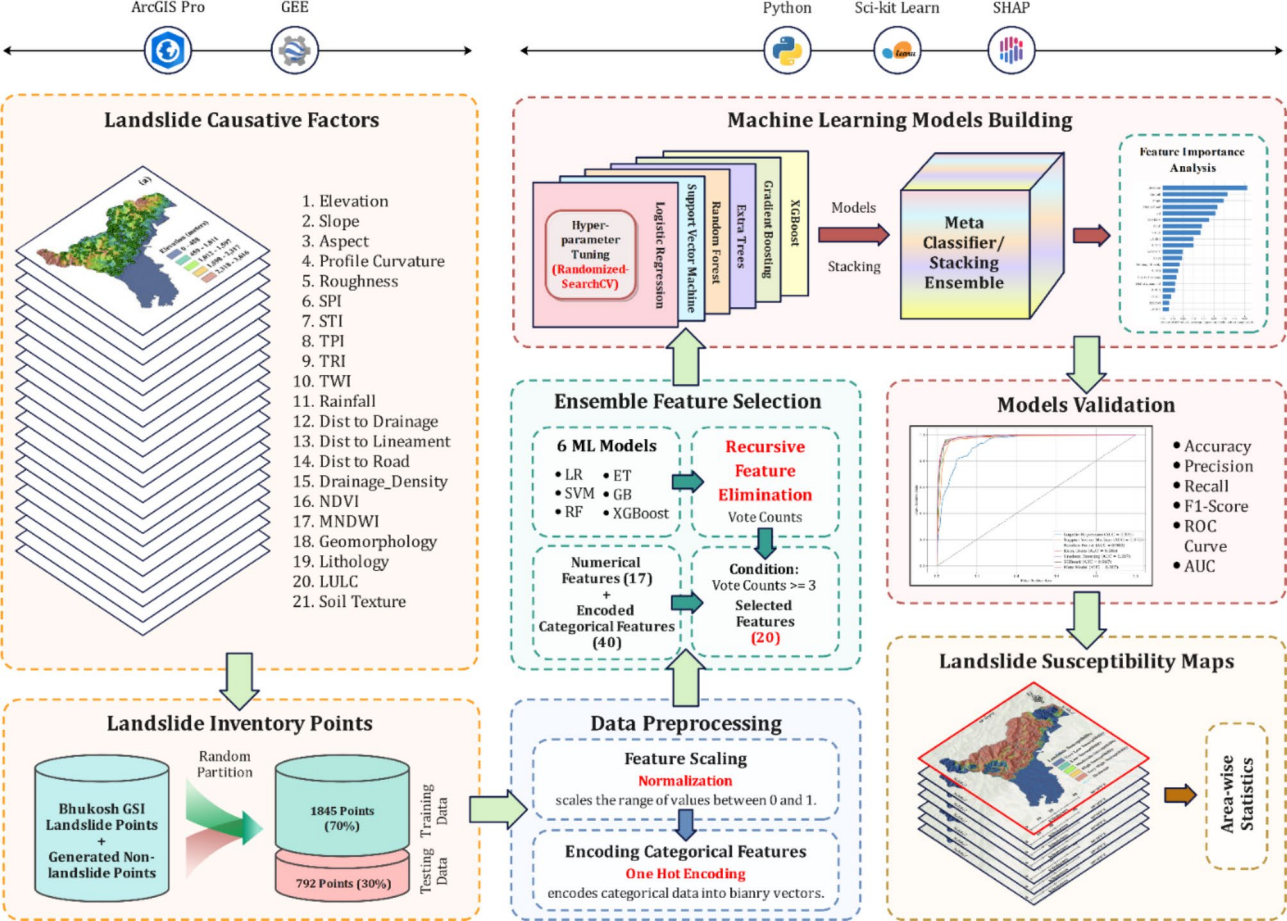
Methodological flow diagram of the present study.

One-hot encoding (OHE) stands as a crucial technique in machine learning for efficiently transforming categorical features into a numerical representation. We applied one-hot encoding to convert categorical features like geology, lithology, LULC, and soil texture into binary vectors. This transformation aligns the features with a format compatible with machine learning algorithms. For example, the lithology variable, which initially has 18 distinct categories, is transformed into a binary vector, where each category is represented by an individual binary column. Subsequently, each observation is assigned a value of ‘1’ in the column that corresponds to its specific lithology category, while all other columns are assigned a value of ‘0’. This procedure generates separate layers for each lithology category, which in turn increases the dimensionality of the dataset. However, by adopting one-hot encoding, the individual layers generated for each category augment the model’s ability to capture the distinct attributes linked to various geological, lithological, land cover, or soil texture types. This leads to a more precise and detailed depiction of the underlying data within the machine learning framework. Despite its efficacy, it’s important to note that the use of one-hot encoding may bring about the “curse of dimensionality,” potentially resulting in large memory and computational demands. The comprehensive methodology of the entire study is visually depicted in the methodological flow diagram, as illustrated in Fig. 3.

### Ensemble feature selection with RFECV

Feature selection is a crucial aspect of machine learning, with the primary purpose of boosting both model performance and interpretability. It becomes imperative, particularly when implementing one-hot encoding, owing to the generation of data with increased dimensionality. The procedure removes less important variables from the dataset while keeping those that carry major importance. Recursive Feature Elimination with Cross-Validation (RFECV) is a powerful methodology that is especially effective in dealing with high-dimensional datasets that have an excess of features.

After implementing one-hot encoding on the four categorical variables, a total of 40 different one-hot encoded features were generated, supplementing the existing set of 17 numerical features. To ensure robust feature selection, we implemented an ensemble recursive feature elimination approach that involves the utilization of six different machine learning models, namely logistic regression (LR), support vector machine (SVM), random forest (RF), extra trees (ET), gradient boosting (GB), and extreme gradient boosting (XGBoost). For each model, RFECV with 5-fold cross-validation was applied, ensuring a thorough evaluation of feature importance and selection across various algorithms and validation sets. The method operates through an iterative process of feature ranking, elimination, and cross-validation. In each iteration, feature importance scores are acquired by the training of the model on the complete collection of features. We opted to eliminate one feature at a time. Following each elimination phase, the model’s performance is rigorously assessed using cross-validation, which includes separating the dataset into folds for training and validation. This recurrent cycle of feature ranking, deletion, and cross-validation continues until a stopping requirement, such as achieving peak model performance, is fulfilled.

### Supervised classification procedure

Following the completion of the feature scaling and feature selection operations, the subsequent essential phase in the machine learning workflow is model development. At this stage, the pre-processed and refined dataset is used to train a prediction model. The choice of the model depends on the nature of the problem and the features of the data. Given the relatively small size of the dataset (1482 data points), deep learning models were deemed unsuitable for this study, as they require large datasets to effectively learn patterns and avoid overfitting^32^. Furthermore, deep learning models are typically seen as “black boxes,” which limits their interpretability- a fundamental criterion in landslide susceptibility mapping, where understanding the influence of individual features is essential.

In contrast, we trained six distinct supervised classification models, including Logistic Regression (LR), Support Vector Machine (SVM), Random Forest (RF), Extra Trees (ET), Gradient Boosting (GB), and Extreme Gradient Boosting (XGBoost). Compared to deep learning models, these machine learning models offer higher levels of interpretability and are especially effective for small to medium-sized tabular datasets^33^. To enhance predictive performance, a meta-classifier was constructed by combining all the individual machine learning models, which often outperform a single ML or DL model^34,35^. The details of the models are included as a Supplementary S2. To find the best set of hyperparameters for each classification task, we employed hyperparameter tuning coupled with a 5-fold cross-validation strategy using scikit-learn’s RandomizedSearchCV class in the Python environment. The selected hyperparameters for all the classifiers are listed in Table 2.

**Table 2:** Hyperparameters of different classifiers selected via RandomizedSearchCV.

Classifier	Hyperparameters	
	Name	Value
Logistic regression	Solver	newton-cg
	penalty	l2
	max_iter	100
	class_weight	None
	C	10
Support vector machine	Kernel	Poly
	gamma	1
	C	10
Random forest	n_estimators	100
	min_samples_split	4
	min_samples_leaf	1
	max_samples	1
	max_features	0.6
	max_depth	None
	criterion	gini
	bootstrap	TRUE
Extra trees	n_estimators	50
	min_samples_split	2
	min_samples_leaf	1
	max_samples	1
	max_features	0.6
	max_depth	None
	criterion	gini
	bootstrap	TRUE
Gradient boosting	subsample	0.7
	n_estimators	150
	min_samples_split	2
	min_samples_leaf	4
	max_features	0.8
	max_depth	None
	learning_rate	0.2
Extreme gradient boosting	subsample	1
	n_estimators	150
	min_child_weight	1
	max_depth	None
	learning_rate	0.3
	gamma	0
	colsample_bytree	1
Meta classifier	Base estimators	Logistic regression
		Support vector machine
		Random forest
		Extra trees
		Gradient boosting
		Extreme gradient boosting
	Final estimator	Logistic regression
	Cross validation	StratifiedKFold(5)

## Evaluation of model performance

### Interpretability of machine learning by Shapley method

Shapley values, derived from cooperative game theory, offer a robust method for interpreting the contribution of each player (in our context, each machine learning model) towards the predictive performance of a coalition (ensemble). This is particularly useful in complex ensemble methods where understanding individual contributions is key to improving overall model performance and transparency.

The Shapley value can be calculated as:

2 $$\:{\varphi\:}_{i}\left(\upsilon\:al\right)=\sum\:_{S\subseteq\:\{{x}_{1},\:\dots\:,\:{x}_{p}\}\backslash\:\left\{{x}_{i}\right\}}\frac{\left|S\right|!\left(p-\left|S\right|-1\right)!}{p!}\left[\upsilon\:al\left(S\cup\:\left\{{x}_{i}\right\}\right)-\upsilon\:al\left(S\right)\right]$$

Where, $\:S$ is a subset of the features used in the model, $\:x$ is the vector of feature values of instance to be explained, $\:p$ the number of features, and $\:\upsilon\:al\left(S\right)\:$is the prediction for feature values in set $\:S$ marginalized over features that are not included in set $\:S$.

### Statistical measures criteria

When validating machine learning algorithms for landslide susceptibility prediction in the Sub-Himalayan region of West Bengal, India, especially in an ensemble Recursive Feature Elimination (RFE) and meta-learning framework, the use of accuracy, precision, recall, and F1 score^36–39^ becomes crucial. Each of these metrics offers unique insights into the performance of the models and understanding them in depth is essential.

#### Accuracy

In the context of landslide susceptibility prediction, accuracy tells us how often the model correctly predicts both landslides and non-landslide events. It is a straightforward measure of the model’s overall effectiveness. However, in regions like the Sub-Himalayan area, where landslide events might be less frequent compared to non-events, high accuracy might not necessarily indicate a highly effective model due to class imbalance Eq. (3).

#### Precision

Precision is particularly important in landslide prediction as it measures the proportion of predicted landslides that were correct. High precision indicates that when the model predicts a landslide, it is likely to be correct. This is crucial in planning and resource allocation, where false alarms (false positives) can be costly and disruptive. In a region prone to landslides, ensuring that the predictions are as precise as possible is key to effective disaster management Eq. (4).

#### Recall

Recall in the context of landslide prediction measures the ability of the model to detect all potential landslide events. High recall is essential as it minimizes the number of missed landslides (false negatives), which is critical for public safety. In the Sub-Himalayan region, where the cost of missing a landslide event could be high in terms of both humans’ lives and infrastructure, a model with high recall is invaluable Eq. (5).

#### F1 score

The F1 Score is a crucial metric in landslide susceptibility modeling as it balances precision and recall, both of which are vital in this context. A high F1 score indicates that the model not only accurately predicts landslides (precision) but also successfully identifies a high percentage of actual landslides (recall). This balance is essential for a reliable landslide prediction model in the Sub-Himalayan region, ensuring both the effective allocation of resources and the safety of the inhabitants Eq. (6).

3 $$\:Precision=\:\frac{TP}{TP+FP}$$

4 $$\:Recall=\:\frac{TP}{TP+FN}$$

5 $$\:F1-Score=\frac{2\left(Precision\times\:Recall\right)}{Precision+Recall}$$

6 $$\:Accuracy=\frac{TP+TN}{TP+FP+TN+FN}\:$$

Where, “TP = True Positive and TN = True Negative are correctly predicted non-landslide points; FP = False Positive, and FN = False Negative are falsely predicted landslide points.

For the ensemble RFE and meta-learning framework applied to landslide susceptibility prediction in the Sub-Himalayan region of West Bengal, these metrics provide a comprehensive assessment of the model’s performance. Accuracy gives a general idea of the model’s effectiveness, precision ensures the reliability of predictions, recall ensures safety by minimizing missed events, and the F1 score provides a balanced view of both precision and recall, indicating the model’s overall efficiency. Considering the diverse and complex terrain of the Sub-Himalayan region, these metrics are crucial in ensuring that the predictions made by the models are not only accurate but also practical and reliable for disaster management and planning purposes.

### Accuracy assessment of the models

Ensuring the accuracy of a model is a critical step in data analysis, and data validation is one of the most important ways to achieve it. Multiple methods are available for validating models, and in this study, ROC (Receiver Operating characteristic) and AUC (Area under the ROC curve) were utilized to verify the accuracy of landslide susceptibility maps^36,40,41^. These maps display the trade-off between sensitivity and specificity. The two-dimensional ROC graph depicts 1-specificity (false positive rate) on the x-axis and sensitivity (true positive rate) on the y-axis. Equations (7), (8) are used to express the attributes of the x and y-axes, where “TN” represents true negative, “FP” represents false positive, “TP” represents true positive, and “FN” represents false negative.

7 $$\:x=1-specificity=1-\left[\frac{TN}{\left(TN+FP\right)}\right]$$

8 $$\:Y=sensitivity=\left[\frac{TN}{(TP+FN)}\right]$$

In addition, the study utilized AUC to quantitatively evaluate the performance of the machine learning methods in the study area^42^. To verify the accuracy of the model, it was compared against survey point data of the study area. The AUC-ROC is a commonly used technique for assessing model performance, with values ranging from 0 to 1, where 1 indicates an excellent relationship between AUC and prediction rate and 0 signifies a poor relationship.

## Results

### Features accuracy and correlation analysis

**Fig. 4:**
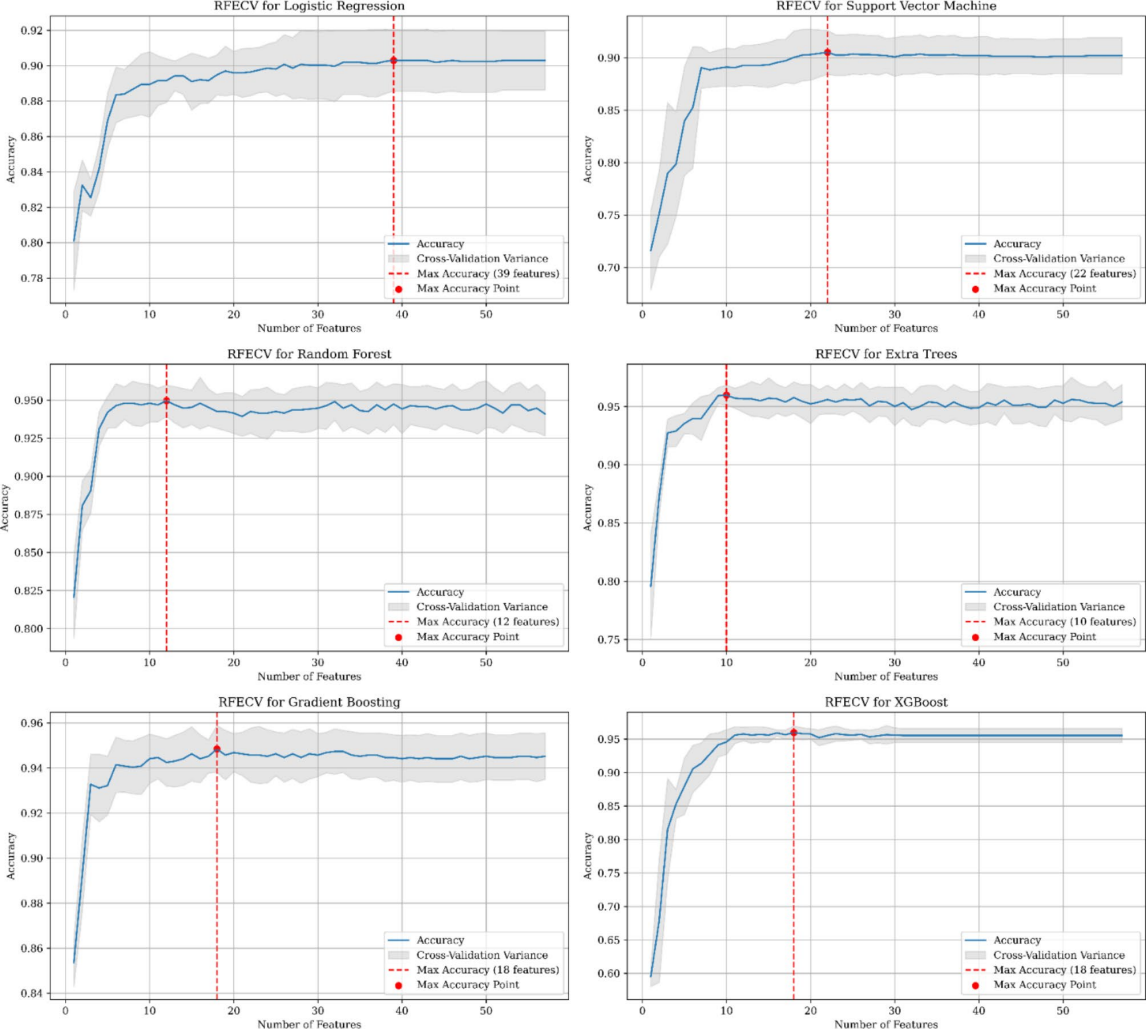
Accuracy vs. number of features selected: Logistic Regression (*LR*), Support Vector Machine (*SVM*), Random Forest (*RF*), Extra Trees (*ET*), Gradient Boosting (*GB*), Extreme Gradient Boosting (*XGBoost*).

Figure 4 illustrates the correlation between the number of features and the corresponding accuracy reached by each model. Notably, LR and SVM revealed a requirement for the maximum number of features, reaching 39 and 22, respectively, to attain their maximal accuracy. In contrast, RF, ET, GB, and XGBoost revealed more efficient feature elimination, demanding only 12, 10, 18, and 18 features, respectively. Remarkably, XGBoost demonstrated the lowest cross-validation variance, suggesting its model stability and consistent performance across diverse feature subsets.

**Fig. 5:**
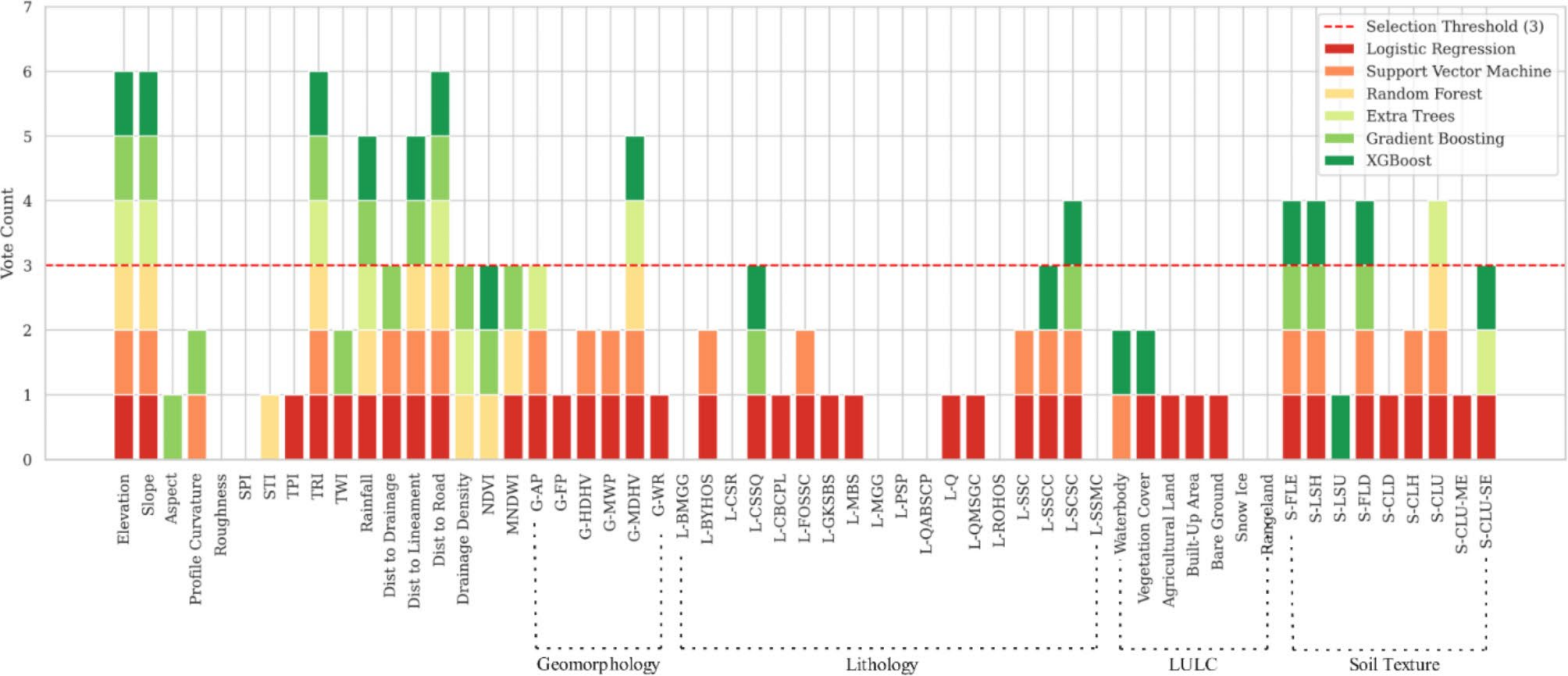
Ensemble vote counts of the numerical and encoded categorical features.

Feature ranking values were extracted for each model, and subsequently, a voting system was established by aggregating the selection values, which could be either ‘TRUE’ or ‘FALSE’. The ultimate selection of features was based on a predefined threshold value of 3, signifying that a feature earned selection status if a minimum of 3 out of the 6 models identified that specific feature as pertinent. Supplementary Table S1 provides a complete overview of feature ranking and the selection status of each feature for individual models. Additionally, it indicates the ensemble vote count and the ultimate selection status. To facilitate readability, given the long names of categorical features, we encoded them into short abbreviations. Figure 5 depicts the relationship between features and total vote counts. Features with a vote count reaching the selection threshold (≥ 3) are considered final selections. A total of 20 features were chosen, containing 10 numerical features and 10 encoded categorical features (Fig. 5). This balanced selection underscores the equal significance accorded to both types of variables within the study.

### Landslide susceptibility zone

**Fig. 6:**
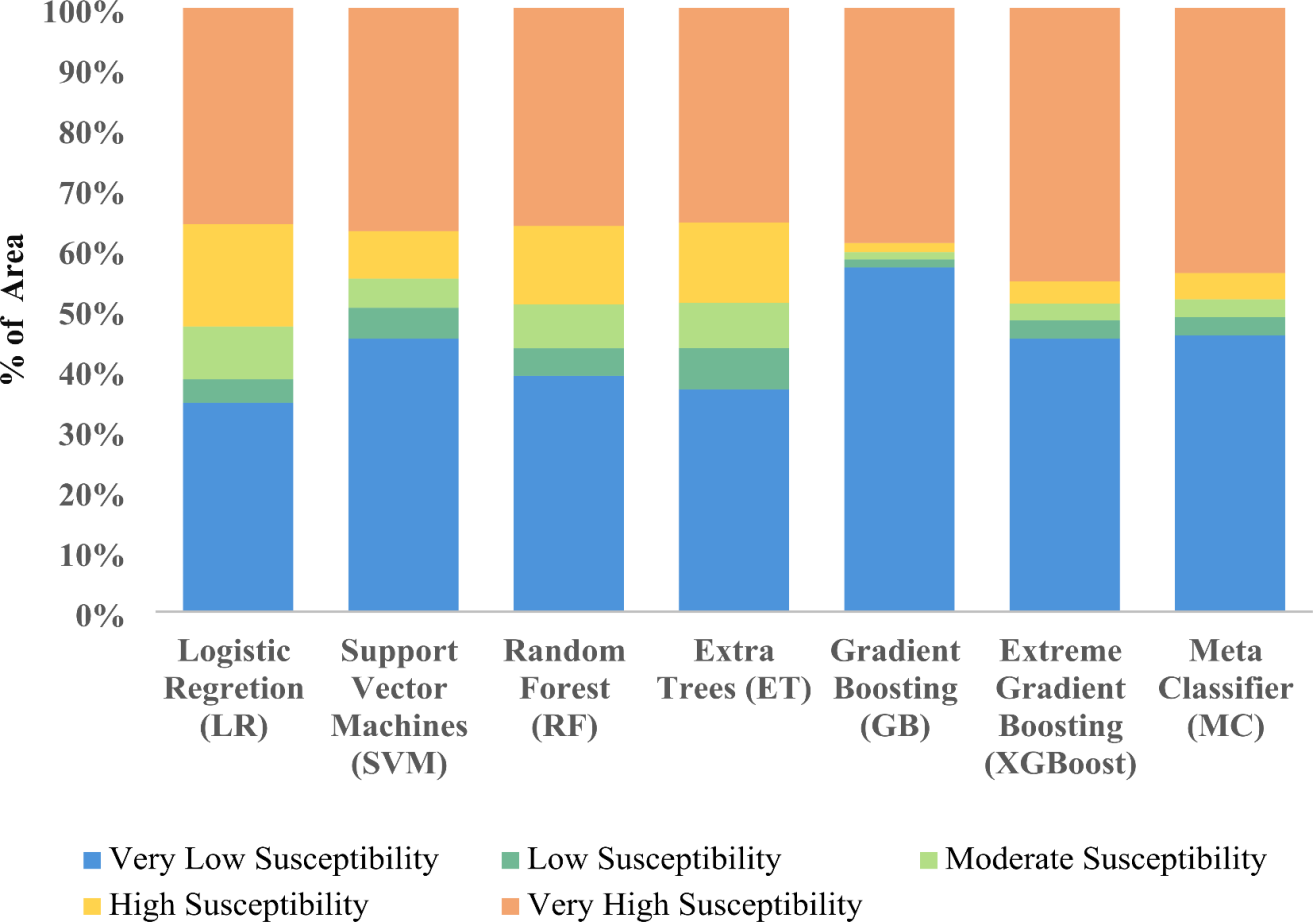
Area-wise proportion of Landslide Susceptibility Class: Logistic Regression (*LR*), Support Vector Machine (*SVM*), Random Forest (*RF*), Extra Trees (*ET*), Gradient Boosting (*GB*), Extreme Gradient Boosting (*XGBoost*), Meta Classifier (*MC*).

**Fig. 7:**
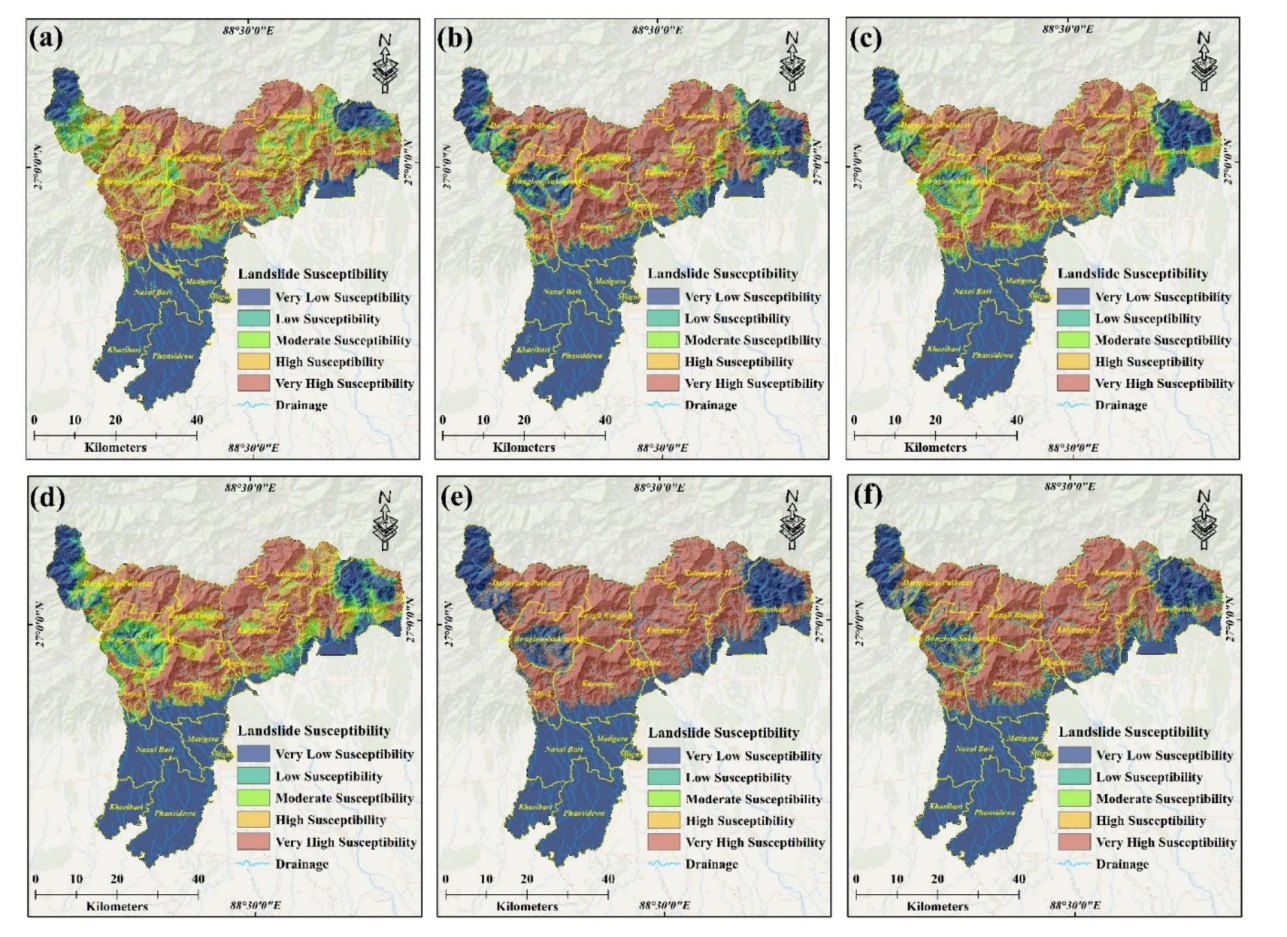
Landslide Susceptibility map: (**a**) Logistic Regression (*LR*), (**b**) Support Vector Machine (*SVM*), (**c**) Random Forest (*RF*), (**d**) Extra Trees (*ET*), (**e**) Gradient Boosting (*GB*), (**f**) Extreme Gradient Boosting (*XGBoost*). [Created by ArcGIS 10.4.1; Esri. (2016). ArcGIS Desktop: Release 10.4.1 [Software]. Environmental Systems Research Institute. https://www.esri.com ].

**Fig. 8:**
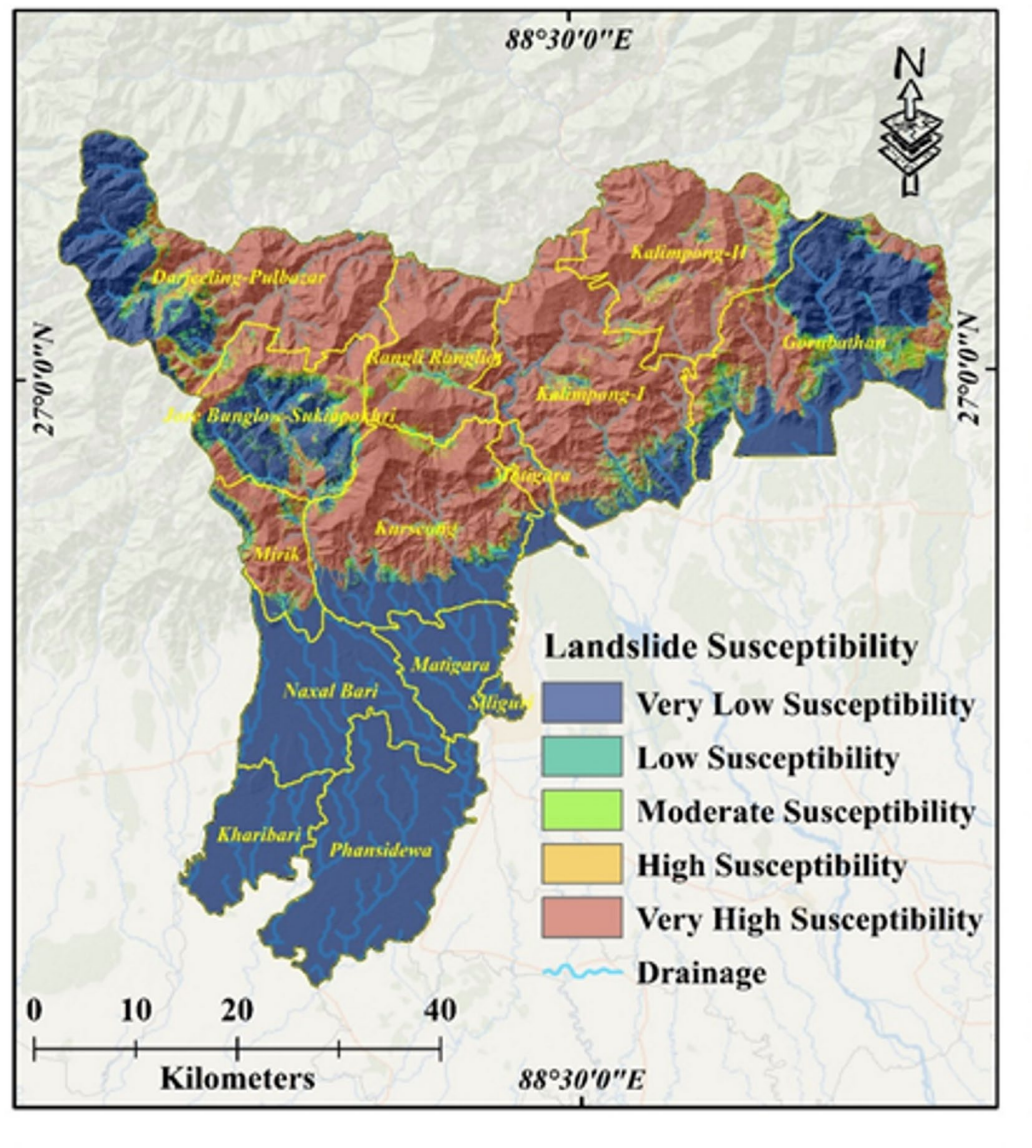
Landslide susceptibility map: Meta classifier (*MC*). [Created by ArcGIS 10.4.1; Esri. (2016). ArcGIS Desktop: Release 10.4.1 [Software]. Environmental Systems Research Institute. https://www.esri.com ].

The current study employed seven advanced machine learning (ML) models, namely Logistic Regression (LR), Support Vector Machine (SVM), Random Forest (RF), Extremely Randomized Trees (ExtraTrees), Gradient Boosting (GB), Extreme Gradient Boosting (XGBoost), and Meta Classifier (MC), to develop landslide susceptibility maps using advanced Remote Sensing (RS) and Geographic Information System (GIS) techniques. Landslide susceptibility was classified into five zones: very low, low, moderate, high, and very high. The performance of each ML model was evaluated based on the area covered (in Km^2^) and the percentage of the total area for each zone (Fig. 6). For Logistic Regression (LR), the distribution across zones was as follows: very low zone 1080.82 Km^2^ (34.61% total area), low zone 120.63 Km^2^ (3.86% total area), moderate zone 273.68 Km^2^ (8.76% total area), high zone 529.86 Km^2^ (16.97% total area), very high zone 1118.2 Km^2^ (35.80% total area). Similarly, the performance metrics for other ML models were documented. For instance, Support Vector Machine (SVM): very low zone 1411.83 Km^2^ (45.20% total area), low zone 159.75 Km^2^ (5.12% total area), moderate zone 151.17 Km^2^ (7.46% total area), high zone 246.14 Km^2^ (13.32% total area), very high zone 1154.29 Km^2^ (35.55% total area). For Random Forest (RF): very low zone 1219.84 Km^2^ (39.06% total area), low zone 142.25 Km^2^ (4.36% total area), moderate zone 94.66 Km^2^ (2.95% total area), high zone 136.085 Km^2^ (4.36% total area), very high zone 1371.59 Km^2^ (43.92% total area). For Extremely Randomized Trees (ExtraTrees): very low zone 1149.88 Km^2^ (36.82% total area), low zone 213.99 Km^2^ (6.85% total area), moderate zone 233.08 Km^2^ (7.46% total area), high zone 415.89 Km^2^ (13.32% total area), very high zone 1110.35 Km^2^ (35.55% total area). For Gradient Boosting (GB): very low zone 1780.39 Km^2^ (57.01% total area), low zone 41.46 Km^2^ (1.33% total area), moderate zone 37.09 Km^2^ (1.19% total area), high zone 48.11 Km^2^ (1.54% total area), very high zone 1216.13 Km^2^ (38.94% total area). For Extreme Gradient Boosting (XGBoost): very low zone 1411.2 Km^2^ (45.18% total area), low zone 95.29 Km^2^ (3.05% total area), moderate zone 86.61 Km^2^ (2.77% total area), high zone 114.69 Km^2^ (3.67% total area), very high zone 1415.39 Km^2^ (45.32% total area), and for Meta Classifier (MC): very low zone 1427.82 Km^2^ (45.72% total area), low zone 95.62 Km^2^ (3.06% total area), moderate zone 92.07 Km^2^ (2.95% total area), high zone 136.08 Km^2^ (4.36% total area), very high zone 1371.59 Km^2^ (43.92% total area) (Table 3). These findings provide valuable insights into the performance and effectiveness of each ML model in landslide susceptibility mapping (Figs. 7, 8).

**Table 3:** Area-wise landslide susceptibility zonation.

Landslide susceptibility zonation	Logistic regression (*LR*)		Support vector machine (*SVM*)		Random forest (*RF*)		Extremely randomized trees (*ExtraTrees*)		Gradient boosting (*GB*)		Extreme gradient boosting (*XGBoost*)		Meta classifier (*MC*)	
	Area in sq. km	Area in (%)	Area in sq. km	Area in (%)	Area in sq. km	Area in (%)	Area in sq. km	Area in (%)	Area in sq. km	Area in (%)	Area in sq. km	Area in (%)	Area in sq. km	Area in (%)
Very low	1080.82	34.61	1411.83	45.20	1219.84	39.06	1149.88	36.82	1780.39	57.01	1411.20	45.18	1427.82	45.72
Low	120.63	3.86	159.76	5.12	142.25	4.55	214.00	6.85	41.47	1.33	95.30	3.05	95.63	3.06
Moderate	273.69	8.76	151.18	4.84	227.07	7.27	233.09	7.46	37.10	1.19	86.62	2.77	92.08	2.95
High	529.86	16.97	246.14	7.88	405.35	12.98	415.89	13.32	48.11	1.54	114.70	3.67	136.09	4.36
Very high	1118.20	35.80	1154.29	36.96	1128.68	36.14	1110.35	35.55	1216.13	38.94	1415.39	45.32	1371.59	43.92
Total area	3123.20	100.00	3123.20	100.00	3123.20	100.00	3123.21	100.00	3123.20	100.00	3123.20	100.00	3123.20	100.00

A very high landslide susceptibility zone lies in a small patch on the northern central part of the Darjeeling hills falling in the Kalimpong subdivision High landslide susceptibility zone lies in Darjeeling Sadar Subdivision on the right side of Teesta River and Kalimpong Subdivision on the left bank of Teesta river. The moderately high landslide susceptibility zone lies northwest-southeast below the high zone in Darjeeling and Kurseong Subdivisions. The moderate landslide susceptibility zone lies in the largest area of the Darjeeling hills where there remains a chance of at least one landslide/km^2^. The low landslide susceptibility zone lies in the northwest corner of the Darjeeling Sadar Sub-division, the southernmost end of the Kurseong Subdivision, and the southern part of the Kalimpong Subdivision (Fig. 7). The occurrence of landslides in this area may be attributed to a combination of factors including a fragile geological composition, intense rainfall, an unstable geological structure, rapid urbanization, and the demand for deforestation and mining activities.

### Model validation

**Fig. 9:**
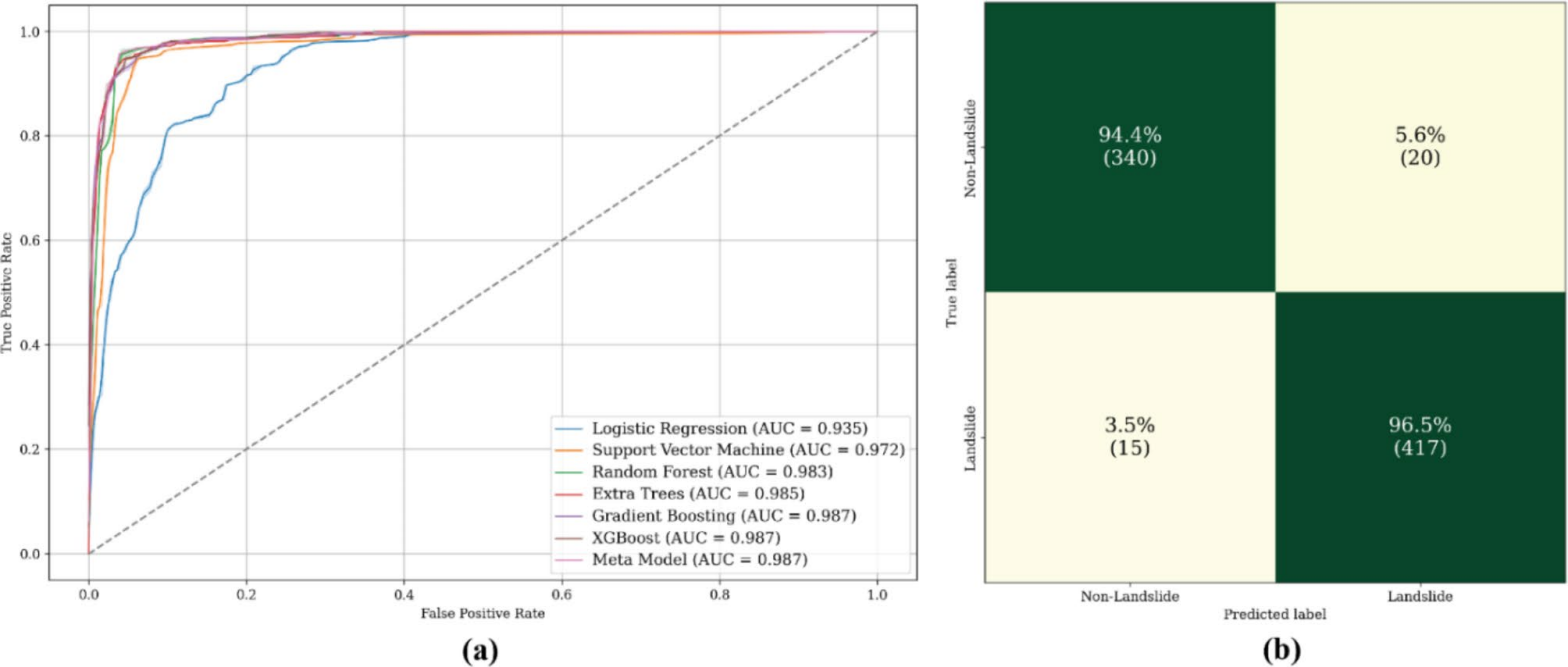
(**a**) Receiver operating characteristic (*ROC-AUC*): Logistic regression (*LR*), Support vector machine (*SVM*), Random forest (*RF*), Extra trees (*ET*), Gradient boosting (*GB*), Extreme gradient boosting (XGBoost), Meta classifier (*MC*) and (**b**) Confusion Matrix.

The performance evaluation of various machine learning models for landslide susceptibility mapping is presented herein. Logistic Regression (LR) demonstrates an accuracy of 0.860, indicating correct predictions in 86% of cases, thus suggesting moderate reliability. Precision stands at 0.824, signifying that 82.4% of landslide predictions are accurate. LR exhibits an exceptional recall of 0.944, capturing 94.4% of actual landslide events. The F1 Score of 0.88 indicates a commendable balance between precision and recall, albeit not the highest among the models. Support Vector Machine (SVM) achieves an accuracy of 0.941, reflecting high reliability with 94.1% correct predictions. SVM exhibits high precision (0.941) and recall (0.951), with an F1 Score of 0.946, indicating a strong balance between precision and recall. Random Forest (RF) achieves an accuracy of 0.943, slightly higher than SVM, suggesting very accurate predictions. RF also demonstrates high precision (0.929) and exceptional recall (0.97), resulting in an F1 Score of 0.949, reflecting a robust balance between precision and recall. ExtraTrees (ET) outperforms other models with an accuracy of 0.946, the highest among the models, along with reliable precision (0.933) and exceptional recall (0.97), resulting in an F1 Score of 0.951, indicating the best balance between precision and recall. Gradient Boosting (GB) achieves an accuracy of 0.943, comparable to RF and SVM, with high precision (0.923) and exceptional recall (0.977), resulting in an F1 Score of 0.949. XGBoost and Meta Classifier (MC) exhibit similar performance metrics to GB, RF, and SVM, demonstrating accuracies of 0.943 and 0.956, respectively (Table 4). All models exhibit strong abilities to differentiate between classes, as evidenced by their respective AUC values The highest AUC values for GB, XGBoost, and MC models were determined to be (0.987), followed by RF (ET (0.985), RF (0.983), SVC (0.972), and LR (0.935) respectively (Fig. 9).

**Table 4:** Model validation metrics.

Landslide susceptibility models	Accuracy	Precision	Recall	F1 score	AUC
Logistic regression	0.860	0.824	0.944	0.880	0.935
Support vector machine	0.941	0.941	0.951	0.946	0.972
Random forest	0.943	0.929	0.970	0.949	0.983
Extra trees	0.946	0.933	0.970	0.951	0.985
Gradient boosting	0.943	0.923	0.977	0.949	0.987
XGBoost	0.943	0.935	0.963	0.949	0.987
Meta classifier	0.956	0.954	0.965	0.960	0.987

## Discussion

The study of landslide prediction is crucial for effective land management, planning, and development in hilly and mountainous areas^43,44,9^. Developing an accurate landslide prediction model is a challenging task. Researchers worldwide have used many methods (including Physically‑based, MCDM, Statistical, Machine Learning, and Deep Learning) and techniques (including Ground-based field study, Complex Mechanical and Engineering, Remote Sensing, and GIS) to create accurate landslide prediction models, but this topic produced disagreement among experts^1,45,13,46,21,5,47^. Consequently, it is necessary to devise and use inventive approaches for constructing and executing landslide prediction models. This study has led to the creation of novel Machine Learning (ML) approaches for analysing landslide prediction. The findings of this study demonstrate the capacity of geospatial techniques in predicting landslides. By using diverse geographic data, such as digital elevation models, satellite images, geological, soil, landcover, and rainfall data, the accuracy of landslide susceptibility may be significantly enhanced. Utilizing machine learning (ML) methods, Logistic Regression (LR), Support Vector Machine (SVM), Random Forest (RF), Extra Trees (ET), Gradient Boosting (GB), Extreme Gradient Boosting (XGBoost), and Meta Classifier (MC), may significantly improve the effectiveness of landslide prediction models. The crucial finding of this study was the use of digital elevation models (DEMs) in predicting landslides. Digital elevation models (DEMs) provide valuable insights into the terrain of a given area, playing a pivotal role in determining the likelihood of landslides. The research determined that the incorporation of DEM data into the prediction model greatly enhanced its performance. Various landslide conditioning factors (LCFs) elevation, slope, slope aspect, profile curvature, roughness, stream power index (SPI), sediment transport index (STI), topographic position index (TPI), topographic ruggedness index (TRI), topographic wetness index (TWI), drainage density, distance to drainage have been generated from DEM data. This emphasizes the significance of precise and high-quality Digital Elevation Models (DEMs) in predicting landslides^48^. Another significant finding was the use of satellite images to predict landslides. The research revealed that the integration of satellite images, particularly Landsat 8, enhanced the accuracy of the prediction model^49^. The reason for this is most likely the capability of satellite imaging to provide data on land cover, vegetation, and water, which are crucial elements in the incidence of landslides^50^. The research determined that the landuse landcover (LULC), normalized difference vegetation index (NDVI), and modified normalised difference water index (mNDWI) were an especially valuable characteristic for predicting landslides^51^. The significance of rainfall data in landslide prediction has also been identified. The research determined that including rainfall data enhanced the efficacy of the prediction model. The increased likelihood of landslides in regions with steep inclines and loose soil is mostly attributed to the significant impact of rainfall. Geological data, including lithology, geomorphology, and lineament analysis, plays a crucial role in landslide prediction by providing valuable information about the terrain and underlying geological conditions. Understanding the geological composition helps in identifying areas with higher susceptibility to landslides based on the stability of the underlying rocks. Certain landforms, such as steep slopes, scarps, or over-steepened hillslopes, are more prone to landslides. Geomorphological mapping helps in identifying these high-risk areas. Analyzing lineaments can help identify zones of weakness in the terrain. Regions with a high density of lineaments may indicate areas where the ground is more susceptible to movement. Assessing the distance from roads helps identify regions where significant cut or fill activities have occurred, providing information about areas with potential slope instability. Soil erosion is a precursor to landslides, and soil texture plays a role in erosion susceptibility. Sandy soils are more prone to erosion because they are easily transported by flowing water, while clayey soils are less susceptible to erosion. Eroded soil can weaken the slope and lead to landslide initiation.

The AUC of the ROC curve was used to validate and compare different machine learning (ML) based landslide models, as well as numerous statistical indicators (Accuracy, Precision, Recall, and F1 Score) to reveal the models’ prediction capability. Models were validated by considering both the training and testing datasets. The results demonstrate that all of the models have strong performance. After conducting a comparative analysis between the results of this study and those of previous studies, the subsequent conclusions were drawn. Although different models exhibit comparable performance levels, their prediction capabilities differ. The outcomes of these single and hybrid machine learning (ML) approaches vary from those of previous studies undertaken globally, although all of them provide a substantial degree of landslide prediction (AUC > 0.850)^52–55,22^. The use of distinct dataset sources by each expert in their inquiry exemplifies the disparity. The disparity in outcomes also arose due to disparities in geographical conditions. The data selection and type, together with the machine learning (ML) algorithm, play a crucial role in determining the outcome. Various modelling approaches might provide different results. In most situations, it was shown that these single or hybrid machine learning (ML) models outperformed standard statistical models or multi-criteria decision-making models in accurately predicting landslides. By comparing the results of the present study to previous research conducted in various study regions with similar topographical and geological conditions, using either single or hybrid models, it can be inferred that there is a disparity in the obtained AUC values and accuracy. Compared with the other methods we found that Random Subspace and Logistic Model Tree (RSLMT) (AUC = 0.815 and Accuracy = 0.738)^56^, Bayesian optimization (BO), namely, Stratified Weighted Averaging (SWA) (AUC = 0.967 and Accuracy = 0.914)^57^, novel ensemble DL model, namely GL-ResNet (Residual Network) (AUC = 0.960 and Accuracy = 0.909)^58^, Random Forest and GBM (AUC = 0.970 and AUC = 0.950)^59^, CatBoost-PSO and CatBoost-GWO (AUC = 0.909 and AUC = 0.910)^50^ the hybrid model used in the current study produced high performances for Sub-Himalayan regions in terms of AUC and overall accuracy (AUC = 0.987 and Accuracy = 0.956). Normally, because of the Meta Classifier (MC) technique, the model can correctly detect the impact of specific predictors even when there is a lot of additive noise in the data. Moreover, the Extremely Randomized Trees (ExtraTrees) (AUC = 0.985 and Accuracy = 0.946), Gradient Boosting (GB) (AUC = 0.987 and Accuracy = 0.943), Extreme Gradient Boosting (XGBoost) (AUC = 0.987 and Accuracy = 0.943) have higher AUC and overall accuracy than other machine learning models used in this study.

As a fundamental statistical model, Logistic Regression (LR) provides a baseline for the prediction of landslide susceptibility. It is effective in modeling binary outcomes (landslide occurrence or non-occurrence) based on a set of predictor variables. In an ensemble framework, LR’s outputs contribute to a foundational understanding of the simpler relationships in the data. Support Vector Machine (SVM) is particularly adept at handling high-dimensional data, which is typical in landslide susceptibility studies due to the multitude of contributing factors. Its capacity to implement different kernel functions allows it to model complex, non-linear relationships between the variables and landslide occurrences, making it a valuable component of the ensemble. Random Forest (RF), an ensemble of decision trees, is known for its high accuracy and ability to handle overfitting. Its strength lies in its capacity to capture non-linear interactions between variables, crucial in complex geographical settings like the Sub-Himalayan region. The model also provides important insights into feature importance, aiding the RFE process. Similar to RF but introducing more randomness in constructing trees, Extra Trees (ET) can capture unexpected patterns in data that other models might miss. This characteristic is particularly useful in unpredictable terrain conditions where landslides occur. Gradient Boosting (GB) builds trees sequentially, with each tree focusing on the errors made by the previous ones. This approach helps in progressively improving model accuracy. In the context of landslide susceptibility, GB can refine predictions, especially in complex landslide scenarios. Extreme Gradient Boosting (XGBoost) provides a more efficient and scalable implementation of GB. Its effectiveness in handling large and complex datasets makes it an invaluable component of the ensemble, particularly given the extensive and varied data involved in landslide susceptibility mapping in the Sub-Himalayan region. The Meta Classifier (MC) integrates the predictions from the individual models, leveraging their respective strengths and compensating for their weaknesses. This integration is central to the meta-learning framework, providing a more accurate and robust prediction of landslide susceptibility than any single model could achieve.

Ensemble Recursive Feature Elimination (RFE) was employed to identify the most relevant features contributing to landslide susceptibility. By iteratively removing the least significant features, RFE optimizes the feature set, enhancing the predictive performance of the models. This process is crucial in a complex geographical setting where numerous factors can influence landslide occurrences. The combined use of different models ensures a comprehensive analysis of the various aspects of landslide susceptibility. This holistic approach improves prediction accuracy. The RFE process helps in narrowing down the most significant predictors of landslides, which is essential for effective risk management and mitigation strategies. The meta-learning framework, through the MC, ensures that the strengths of individual models are harnessed effectively, leading to a synergistic improvement in landslide prediction. The application of an ensemble RFE and meta-learning framework incorporating multiple advanced machine-learning models presents a significant advancement in predicting landslide susceptibility in the Sub-Himalayan region of West Bengal, India. This comprehensive approach not only improves prediction accuracy but also offers crucial insights for effective landslide risk management and planning in this geologically sensitive region.

**Fig. 10:**
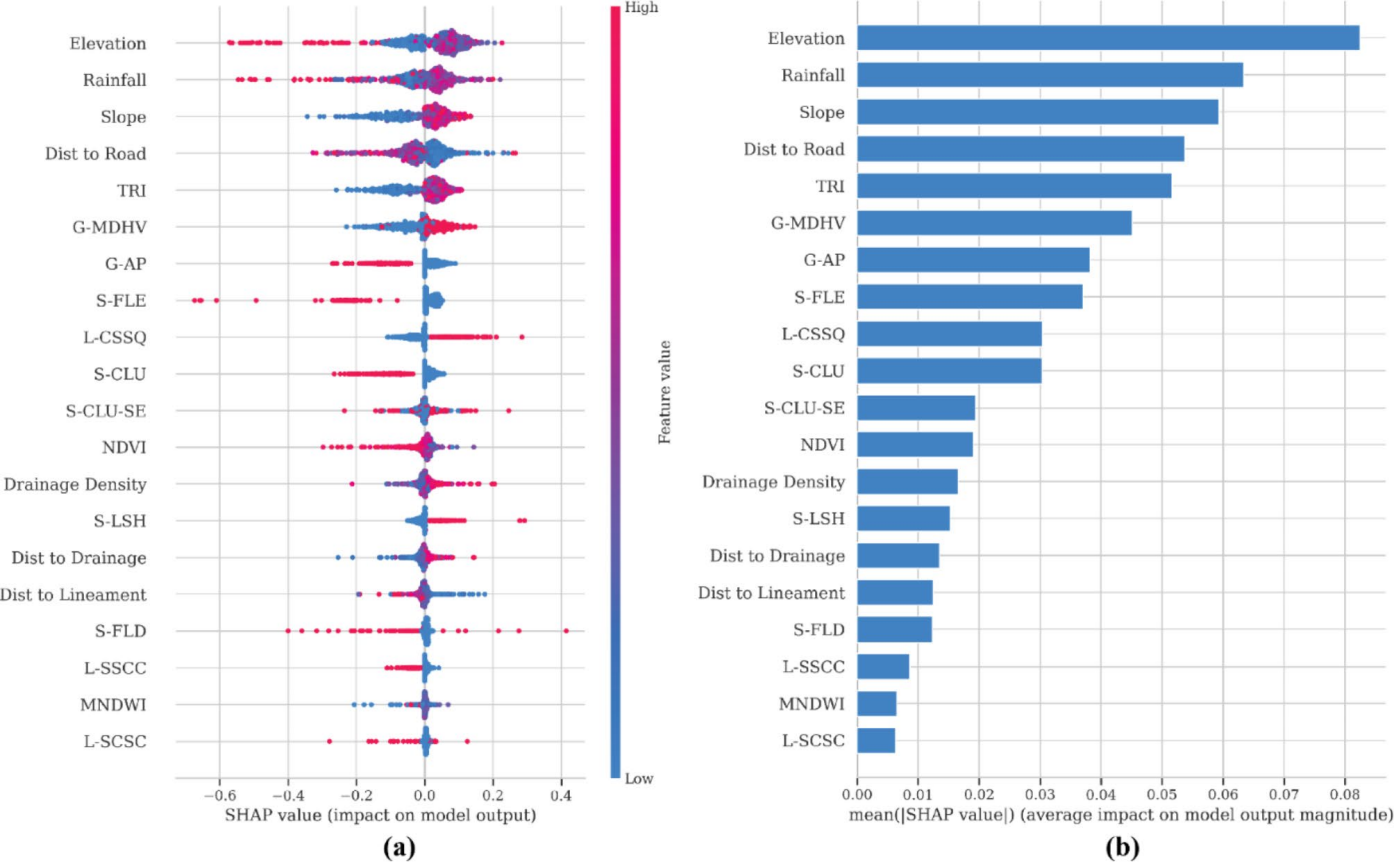
(**a**) Shapley value of all sample features and (**b**) Feature Importance Graph.

We also explored the importance of individual features in our modeling approach, aiming to discern their relative significance. Given the diverse array of variables encompassing climate, geomorphology, lithology, land use, land cover (LULC), and soil texture, it’s evident that they don’t hold equal sway or uniform importance in forecasting landslides. Thus, it’s crucial to identify the most impactful features, allowing domain experts to grasp pivotal factors that overshadow others in importance. In this study, we employed SHAP (SHapley Additive exPlanations) as a post-hoc method to gain insights into prediction outcomes and pinpoint landslide-influencing factors contributing significantly to model outputs. Features with larger or smaller Shapley values indicate a proportionally higher or lower influence on the predicted areas susceptible to landslides. To gauge their global importance, we calculated the average absolute Shapley value per feature across the dataset. The outcomes, depicted in Fig. 10, highlight red bars symbolizing features positively associated with landslide occurrence, thereby exerting a favorable influence on the predicted landslide-susceptible areas. From Fig. 10a and b, it’s evident that the top five most crucial variables/features were Elevation (mean SHAP value > + 0.08), followed by Rainfall (mean SHAP value > + 0.06), Slope (mean SHAP value > + 0.05), Dist to Road (mean SHAP value > + 0.05), and TRI (mean SHAP value > + 0.05). The prominence of Elevation as the most critical feature in predicting landslide susceptibility can be attributed to the vital terrain information it provides regarding slope and aspect. Steep slopes are predisposed to landslides due to their diminished stability and heightened susceptibility to gravity-induced soil and rock movement. To mitigate the impacts and minimize the damage caused by such a catastrophe, many strategies may be used, such as implementing land-use planning to prevent development in regions prone to high risks. Engineering procedures such as the construction of retaining walls, installation of drainage systems, implementation of slope stabilisation techniques, Implementation of early warning systems for the detection and prediction of prospective landslides, consistent monitoring of slopes, soil, and weather conditions, and the creation of hazard maps to identify regions with a high risk of landslides. Swift and efficient reaction, together with a comprehensive recovery strategy, aimed at minimizing damage and assisting communities in their recovery efforts.

## Conclusion and outlook

Our research focuses on enhancing landslide susceptibility prediction in the Sub-Himalayan region of West Bengal, India, representing a significant advancement in geospatial predictive modelling. By employing cutting-edge machine learning techniques, we developed a robust framework to better understand and forecast landslides in a region marked by complex topography and diverse environmental influences. Logistic Regression (LR) served as a baseline, offering insights into simple, linear relationships within the data. Support Vector Machine (SVM) excelled in handling the problem’s high-dimensional and nonlinear characteristics. The Random Forest (RF) and Extra Trees (ET) methods effectively captured intricate, nonlinear dependencies while demonstrating resilience against overfitting. Gradient Boosting (GB) and Extreme Gradient Boosting (XGBoost) further improved predictive accuracy by addressing the limitations of earlier models. Our Meta Classifier (MC) ensemble integrated these diverse methods, combining their individual strengths to achieve superior predictive performance and a comprehensive understanding of landslide dynamics. Additionally, we leveraged ensemble Recursive Feature Elimination (RFE) to optimize feature selection, enhancing model effectiveness in the geologically challenging Sub-Himalayan terrain.

This study highlights the importance of an integrative approach where machine learning complements traditional predictive methodologies rather than functioning in isolation. Despite achieving high accuracy, challenges remain in interpreting complex models. Tools like SHAP (SHapley Additive exPlanations) offered valuable post-hoc interpretability, but further advancements are necessary to bridge the gap between predictive insights, causal reasoning, and practical applications. Future efforts should prioritize the inclusion of richer geospatial datasets, such as those derived from Lidar and InSAR technologies, to further refine predictive capabilities. Moreover, future research should examine the impact of climate change on landslide dynamics by incorporating factors like increased precipitation intensity and land-use changes into predictive models. This adaptation will enhance their relevance for addressing climate-driven risks. Cross-regional validation is essential to test the framework’s scalability and adaptability across diverse terrains, soil types, and climatic conditions, ensuring broader applicability. Integrating physical-based models with machine learning can improve accuracy and transparency by combining data-driven insights with physical processes. Additionally, incorporating citizen-generated data on landslide events can refine models, particularly in under-monitored areas, while fostering community engagement and resilience. These advancements will make landslide prediction frameworks more robust, interpretable, and globally applicable. Developing models that strike a balance between accuracy and transparency will also be vital for meeting the scientific and policy needs of stakeholders.

The global implications of this work are substantial. Landslides pose significant threats in many geologically vulnerable areas, and our framework offers a scalable and adaptable solution to similar challenges worldwide. By advancing predictive capabilities, this research supports more informed land-use planning, efficient resource allocation, and proactive disaster management. The integration of ensemble learning and feature optimization presents a novel methodology that can be extended to other regions with environmental and geospatial complexities. Ultimately, our study deepens the scientific understanding of landslide dynamics and establishes a precedent for combining machine learning with interdisciplinary insights to address critical global challenges.

## Electronic supplementary material

Below is the link to the electronic supplementary material.

Supplementary Material 1

## Data Availability

The datasets generated during and/or analysed during the current study are available from the corresponding author on reasonable request.
