# Publisher Correction: Improving landslide susceptibility prediction through ensemble recursive feature elimination and meta-learning framework

**DOI:** 10.1038/s41598-025-06242-z

**Published:** 2025-06-12

**Authors:** Krishnagopal Halder, Amit Kumar Srivastava, Anitabha Ghosh, Subhabrata Das, Santanu Banerjee, Subodh Chandra Pal, Uday Chatterjee, Dipak Bisai, Frank Ewert, Thomas Gaiser

**Affiliations:** 1 Leibniz Centre for Agricultural Landscape Research (ZALF), Eberswalder Strasse 84, 15374 Müncheberg, Germany; 2 Institute of Crop Science and Resource Conservation, University of Bonn, 5D-53115 Katzenburgweg, Bonn, Germany; 3 Coastal Environmental Studies Research Centre, Egra S.S.B. College, (Affiliated to Vidyasagar University), Kharagpur, 721429 West Bengal India; 4 Langmuir Center of Colloids and Interfaces, Columbia University in the City of New York, New York, 10027 USA; 5 College of Agriculture, Chhatrapati Shahu Ji Maharaj University, Kanpur, 208012 UP India; 6 Department of Geography, The University of Burdwan, Barddhamān, 713104 India; 7 Department of Geography, Bhatter College, Dantan, Kharagpur, 721426 West Bengal India

Correction to: *Scientific Reports* 10.1038/s41598-025-87587-3, published online 12 February 2025

In the original version of the Article, Figure 8 was a duplication of Figure 7.

The original Article has been corrected.

