# Supplementary material for: Improving landslide susceptibility prediction through ensemble recursive feature elimination and meta-learning framework
Source: Sci Rep. 2025 Feb 12;15:5170. doi: 10.1038/s41598-025-87587-3 (PMC11822027; doi:10.1038/s41598-025-87587-3)
Supplement: Supplementary file 1 — Supplementary Material 1 [file 41598_2025_87587_MOESM1_ESM.docx]

**Supplementary S1: Landslide Susceptibility Parameters (LSP)**

***Landslide Susceptibility Parameters (LSP)***

**(a) Elevation (Ele):** Elevation is a fundamental factor in landslide occurrence. Higher elevations are often associated with steeper slopes and more varied topography, which can predispose areas to landslides. Elevation changes can affect climatic conditions, such as precipitation patterns and temperature, influencing soil moisture and vegetation cover. In mountainous regions, higher elevations may be more susceptible to freeze-thaw cycles, leading to soil weakening and increased landslide risk. The elevation of the study area ranges from 18 to 3601 meters (Figure S1 a). The elevation map of the study area has been prepared based on ASTER GDEM (30m resolution) and classified by natural breaking in ArcGIS 10.4.1 as 18 – 445 meters, 446 – 998 meters, 999 - 1,583 meters, 1,584 - 2,300 meters, and 2,301 - 3,601 meters.

**(b) Slope (Sl):** Slope is directly correlated with landslide probability. Steeper slopes have a higher potential for landslides due to the gravitational force acting on them. The stability of a slope is determined by its angle; as the slope angle increases, the downslope force increases, reducing stability. Slope material composition and internal structure also play critical roles in determining slope stability. The filled DEM was used for slope analysis and using the “Slope” function under the “Surface” function of “Spatial Analyst Tools” in the ArcGIS 10.4.1 platform a slope map was generated for the study area. The slope angle of the district ranges from 0.00° to 69.54° (Figure S1 b). Flat (0.00° - 9.27°), gentle (9.28° - 18.54°), moderate (18.55° - 27.00°), moderately steep (27.01° - 36.27°), steep (36.28° - 69.54°) slopes.

**(c) Slope Aspect (SA):** Slope aspect refers to the direction a slope faces. It influences microclimatic conditions, such as sunlight exposure and wind patterns, which in turn affect soil moisture and vegetation growth. North-facing slopes in the Northern Hemisphere, for example, tend to be cooler and wetter, potentially leading to different landslide susceptibilities compared to south-facing slopes. The slope aspect map was generated from filled ASTER GDEM and using the “Aspect” function under the “Surface” function of “Spatial Analyst Tools” in the ArcGIS 10.4.1 platform. It is shown in ten classes of the respective area (Figure S1 c). Flat (-1), North (0-22.5), Northeast (22.5-67.5), East (67.5-112.5), Southeast (112.5-157.5), South (157.5-202.5), Southwest (202.5-247.5), West (247.5-292.5), Northwest (292.5-337.5), North (337.5-360).

**(d) Profile Curvature (PC):** Profile curvature affects the acceleration and deceleration of flow across a surface. Convergent profile curvature can lead to increased soil saturation, as water accumulates in the concave areas of a slope. Conversely, convex areas may have better drainage and less water accumulation, potentially reducing landslide risk. The profile curvature map was generated from filled ASTER GDEM and using the “Curvature” function under the “Surface” function of “Spatial Analyst Tools” in the ArcGIS 10.4.1 platform. Profile curvature has been classified into concave (-14.69 - 0.00), flat (0.01), and convex (0.02 - 11.63) (Figure S1 d).

**(e) Roughness (Rn):** Terrain roughness is indicative of surface irregularity and can influence water runoff and sediment transport. Rougher surfaces with more irregularities can trap more water and sediment, potentially increasing the weight on a slope and the likelihood of landslides. Smooth surfaces allow easier water flow, which can reduce saturation-related landslide risk. The roughness of the region is calculated from the filled ASTER GDEM using the “Focal Statistics” function under “Neighborhood” and the “Raster Calculator” function under the “Map Algebra” function of “Spatial Analyst Tools” in the ArcGIS 10.4.1 platform.

$$Roughness= \frac{(FSmean -FSmin)}{(FSmax-FSmin)} (1)$$

Where,$FSmean$ is the mean elevation of the defined region, 𝐹𝑆𝑚𝑎𝑥 and 𝐹𝑆𝑚𝑖𝑛 are the minimum and maximum elevation of that region respectively. Roughness values range from 0.11 to 0.89 (Figure S1 e). Roughness has been classified into: 0.11 - 0.39, 0.40 - 0.46, 0.47 - 0.53, 0.54 - 0.60, and 0.61 - 0.89.

**(f) Stream Power Index (SPI):** SPI is a measure of the erosive power of flowing water. High SPI values indicate a greater potential for stream erosion, which can undermine slope stability by removing support material at the base of slopes. This process can lead to increased susceptibility to landslides, particularly in areas near active watercourses. The SPI of the region is calculated from the filled ASTER GDEM using the “Hydrology” function and “Raster Calculator” function under the “Map Algebra” function of “Spatial Analyst Tools” in the ArcGIS 10.4.1 platform.

$$SPI=Ai \times tan\beta(2)$$

Where, “𝐴i is the contributing catchment area and 𝛽 is the slope angle”. SPI values range from 0.00 to >498 (Figure S1 f). SPI has been classified into: 0.00 - 0.01, 0.02 – 100, 100 – 193, 193 – 498, and >498.

**(g) Sediment Transport Index (STI):** STI assesses the potential for sediment movement and deposition. High STI values suggest that an area is prone to receiving or transporting large amounts of sediment, which can impact slope stability. Sediment accumulation can add weight to slopes and change their angle, affecting their susceptibility to landslides. The SPI of the region is calculated from the filled ASTER GDEM using the “Hydrology” function and “Raster Calculator” function under the “Map Algebra” function of “Spatial Analyst Tools” in the ArcGIS 10.4.1 platform.

$$STI= \left[ \frac{\left( \frac{F_{a}}{\delta x} \right)^{2}}{\left( \frac{Sig n (S_{a)}}{\delta y} \right)^{2}} \right] (3)$$

Where, $F_{a}$represents the flow accumulation and $S_{a}$ represents the slope raster, derived from ASTER GDEM, and $\delta x$ and $\delta y$ represents the constant. STI values range from 0.00 to >22.15 (Figure S1 g). STI has been classified into: 0.00 - 0.01, 0.02 - 3.69, 3.70 - 11.07, 11.08 - 22.14, >22.15.

**(h) Topographic Position Index (TPI):** TPI measures the relative position of landforms within the surrounding landscape. It helps in identifying ridges, valleys, and slopes. Positions such as valley bottoms may accumulate more moisture, leading to soil saturation and increased landslide risk, whereas ridgelines might be less prone to such occurrences. The TPI map of the study area was derived by applying the “Topographic Position Index” function of the “Land Facet Corridor Designer Tool” in the ArcGIS 10.4.1 platform.

$$TPI=Z_{0}-\tilde{Z}_{i} (4)$$

$$\tilde{Z}_{i}= \frac{1}{n_{R}}\sum_{i\in R} Z_{i} (5)$$

Topographic Position Index (TPI) illustrates the variation in altitude of a specific location ($Z_{0}$) compared to the average altitude ($\tilde{Z}$) within a certain distance (R). TPI values range from -3.14 to 5.29 (Figure S1 h). TPI has been classified into: -3.14 - 5.29. -3.14 - -0.49, -0.48 - -0.03, -0.02 - 0.43, 0.44 - 1.13, and 1.14 - 5.29.

**(i) Topographic Ruggedness Index (TRI):** TRI quantifies the amount of elevation difference between adjacent cells of a digital elevation model. High TRI values indicate a very rough and irregular terrain, which is often associated with varied and unstable geological structures, predisposing these areas to landslides. The TRI of the region is calculated from the filled ASTER GDEM using the “Focal Statistics” function under “Neighborhood” and the “Raster Calculator” function under the “Map Algebra” function of “Spatial Analyst Tools” in the ArcGIS 10.4.1 platform.

$$TRI=\sqrt{Abs({max}^{2} - {min}^{2})} (6)$$

Here, 𝑚𝑎𝑥 and 𝑚𝑖𝑛 are the maximum and minimum elevation of the defined region respectively. TPI values range from 0 to 851 (Figure S1 i). TRI has been classified into 0 – 851. 0 – 110, 111 – 220, 221 – 317, 318 – 424, and 425 – 851.

**(j) Topographic Wetness Index (TWI):** TWI is a measure of soil moisture potential. It is based on the assumption that water flows down slopes, accumulating in lower areas. Higher TWI values usually correlate with greater soil moisture, which can weaken soil strength and increase the risk of landslides. The TWI of the region is calculated from the filled ASTER GDEM using the “Hydrology” function and “Raster Calculator” function under the “Map Algebra” function of “Spatial Analyst Tools” in the ArcGIS 10.4.1 platform.

$$TWI=Ln\left( \frac{a}{\tan\beta} \right) (7)$$

Where *α* is the upslope contributing area and *tan β* is the slope angle. Likewise, here = $\frac{A}{L}$, Amongst them, 𝐴 and 𝐿 denote the total basin area and length of the contour. TWI values of the studied area varied from 2.53 to 28.64 (Figure S1 j). TWI has been classified into: 2.53 - 8.17, 8.18 - 12.26, 12.27 - 14.72, 14.73 - 18.40, and 18.41 - 28.64

**(k) Drainage Density (DD):** Drainage density reflects the extent of stream networks within a given area. High drainage density can indicate a greater potential for surface runoff and erosion, which may contribute to slope instability. This factor is particularly significant in regions with frequent heavy rainfall events. The drainage density map was prepared using the “Line Density” function under the “Density” function of “Spatial Analyst Tools” in the ArcGIS 10.4.1 platform.

$$Dd= \sum_{i=1}^{i=n} \frac{D_{i}}{A}\left( {km}^{-1} \right) (8)$$

Where, ${"D}_{i}$is the total length of the stream in the grid (km) and $A$ is the grid area (km^2^)”. The drainage density of the area ranges from 0.00 to 0.80 km/sq. km (Figure S1 k). The study area has been divided into several drainage density classes: 0.00 - 0.18 km km^-2^, 0.19 - 0.30 km km^-2^, 0.31 - 0.42 km km^-2^, 0.43 - 0.54 km km^-2^, and 0.55 - 0.80 km km^-2^.

**(l) Distance to Drainage (DTD):** Proximity to drainage channels can be a critical factor in landslide susceptibility. Areas close to drainage networks may experience more undercutting and erosion at the base of slopes, reducing slope stability and increasing the likelihood of landslides. The Distance to drainage map was prepared using the “Euclidean Distance” function under the “Distance” function of “Spatial Analyst Tools” in the ArcGIS 10.4.1 platform. Distance to drainage ranges from 0 to 3,691 meters (Figure S1 l). Distance to drainage values has been classified into 0 – 400 m, 401 – 800 m, 801 - 1,200 m, 1,201 - 1,600 m, 1,601 - 3,691m.

**(m) Distance to Lineament (DTL):** Lineaments are natural linear features like faults and fissures. Proximity to these features can indicate zones of structural weakness in the Earth's crust. Areas near lineaments may have increased fracture density in rock masses, leading to a higher susceptibility to landslides. The Distance to Lineament map was prepared using the “Euclidean Distance” function under the “Distance” function of “Spatial Analyst Tools” in the ArcGIS 10.4.1 platform. Distance to lineament ranges from 0 to 10,000 meters (Figure S1 m). Distance to lineament values has been classified into 0 - 2,000 meters, 2,001 - 4,000 meters, 4,001 - 6,000 meters, 6,001 - 8,000 meters, and 8,001 - 10,000 meters.

**(n) Distance to Road (DTR):** The construction and maintenance of roads can significantly alter landscape stability. Roads can change natural drainage patterns and increase erosion, particularly if they are built on slopes. The distance from roads can be an important factor in assessing landslide risk, as areas closer to roads might be more affected. The Distance to the Road map was prepared using the “Euclidean Distance” function under the “Distance” function of “Spatial Analyst Tools” in the ArcGIS 10.4.1 platform. Distance to roads ranges from 0 - 13,031 meters (Figure S1 n). Distance to road values has been classified into 0 - 2,600 meters, 2,601 - 5,200 meters, 5,201 - 7,800 meters, 7,801 - 10,000 meters, and 10,001 - 13,031 meters.

**(o) Rainfall (RF):** Intense and prolonged rainfall is a primary trigger for landslides. Rainfall contributes to increasing soil moisture and pore-water pressure, weakening slope materials. Areas with high rainfall are often at a greater risk, especially if the rain is intense or prolonged. The rainfall map was prepared using the “Kriging” function under the “Interpolation” function of “Spatial Analyst Tools” in the ArcGIS 10.4.1 platform. Rainfall values vary from 1,683 – 2688 mm (Figure S1 o). Rainfall values have been classified into 1,683 - 1,888 mm, 1,889 - 2,037 mm, 2,038 - 2,195 mm, 2,196 - 2,392 mm, and 2,393 - 2,688 mm.

**(p) Normalised Difference Vegetation Index (NDVI):** NDVI is a measure of plant health and density. Dense vegetation can help stabilize slopes by reinforcing soil and absorbing water, reducing landslide risk. Conversely, areas with sparse vegetation might have less protection against erosion and landslides. The NDVI was computed using the “Raster Calculator” function under the “Map Algebra” function of “Spatial Analyst Tools” in the ArcGIS 10.4.1 platform.

$$NDVI= \frac{NIR-Red}{NIR+Red'} (9)$$

Where, “$Red$signifies red band and $NIR$ signifies near-infrared band”. NDVI values of the district range from -0.12 to 0.73 (Figure S1 p). NDVI values have been classified into: -0.12 - 0.15, 0.16 - 0.25, 0.26 - 0.32, 0.33 - 0.38, and 0.39 - 0.73.

**(q) Modified Normalised Difference Water Index (mNDWI):** mNDWI is used to monitor changes in water content in the soil and vegetation. High mNDWI values can indicate increased soil moisture, which may lead to a higher risk of landslides, especially in areas where soil cohesion is reduced. The mNDWI was computed using the “Raster Calculator” function under the “Map Algebra” function of “Spatial Analyst Tools” in the ArcGIS 10.4.1 platform.

$$mNDWI= \frac{Green-MIR}{Green+MIR'} (10)$$

Where, “$Green$ signifies green band and $MIR$ signifies middle infra-red band”. mNDWI values of the study area range from -0.69 to 0.20 (Figure S1 i). mNDVI values are -0.69 - -0.21, -0.2 - -0.16, -0.15 - -0.11, -0.1 - 0.01, and 0.02 - 0.20.

**(r) Landuse Landcover (LULC):** Different land uses and cover types can significantly impact landslide susceptibility. Urban areas with impermeable surfaces may have increased runoff, while deforested areas may lack the root structures necessary to stabilize slopes. Natural vegetation areas tend to offer more protection against landslides. The supervised classification technique has been adopted in ERDAS Imagine and ArcGIS software to prepare the LULC map (Figure S1 r). Classified zones are Waterbody (0.61%), Vegetation Cover (71.37%), Agricultural Land (10.61%), Built-Up Area (9.68%), Bare Ground/Snow/Ice (1.56%), Rangeland (6.17%).

**(s) Geomorphology (GM):** Geomorphological features reflect the Earth's surface processes and materials. Certain geomorphological settings, such as steep, unstable slopes or weak geological materials, are more prone to landslides. Understanding the geomorphology of an area can provide insights into potential landslide hazards. The lithological map of the entire district has been classified into five distinctive zones based on BHUKOSH GSI (Figure S1 s). Classified zones are Alluvial Plain (17.80%), Flood Plain (10.60%), Highly Dissected Hills and Valleys (30.07%), Moderately Dissected Hills and Valleys (38.27%), Waterbody – River (3.25%).

**(t) Soil Texture (ST):** The texture of the soil, determined by the size of its particles, affects its water retention and drainage properties. Coarser soils generally have better drainage and are less prone to landslides, while fine-grained soils can retain more water, increasing landslide risk. The soil texture map of the entire study area has been classified into nine distinctive zones based on NBSSLUP (Figure S1 s). Classified zones are Loamy Skeletal (Lithic Udorthents) (7.48%), Coarse Loamy (Typic Udorthents, severe erosion) (38.65%), Fine Loamy (Umbric Dystrochrepts) (6.43%), Loamy-Skeletal (Typic Haplumbrepts) (12.62%), Coarse Loamy (Typic Udorthents, moderate erosion) (2.96%), Coarse Loamy (Umbric Dystrochrepts) (6.88%), Fine Loamy (Fluventic Eutrochrepts) (6.06%), Coarse Loamy (Typic Haplaquents) (1.42%), Coarse Loamy (Aquic Uditluvents) (17.46%).

**(u) Lithology (LITH):** Lithology refers to the physical characteristics of rock types. Certain rock types, like shale and clay, are more susceptible to weathering and erosion, leading to higher landslide risks. Understanding the lithology of an area is crucial in assessing its landslide susceptibility. The lithological map of the entire district has been classified into twenty-one distinctive zones based on BHUKOSH GSI (Figure S1 u). Classified zones are Banded Migmatite, Garnet Bt Gneiss (37.289%), Mica Schist, Boulder Slate, Conglomerate, Phyllite (0.116%), Brown and Yellowish Colour Highly Oxidized Soil (7.475%), Calc Silicate Rock (0.049%), Chlorite Sericite Schist and Quartzite (11.382%), Compact Boulders, Cobbles, Pebbles with Latosol (0.247%), Dolimitic Quartzite, Chert, Phyllite, Slate (0.010%) etc.


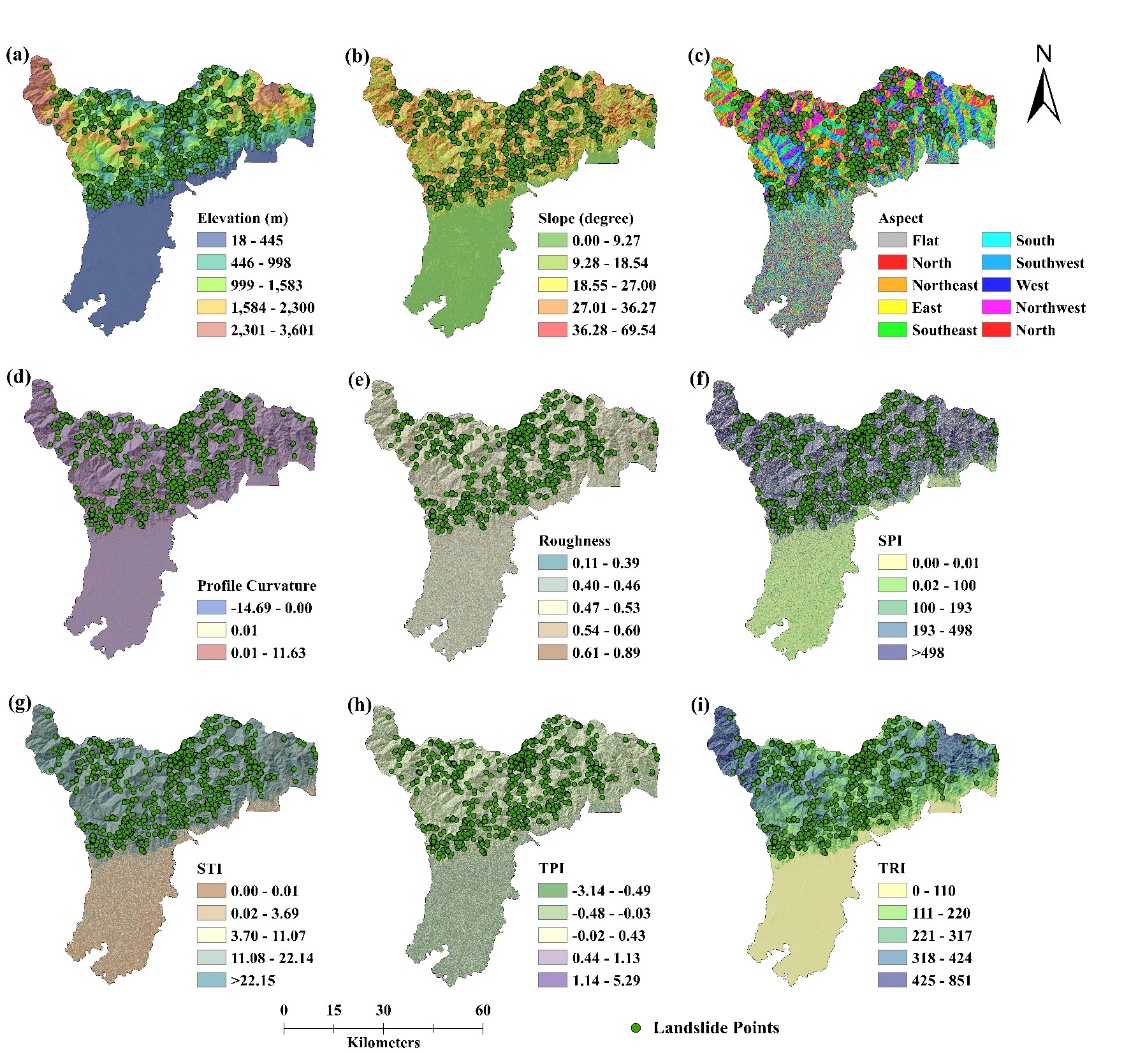


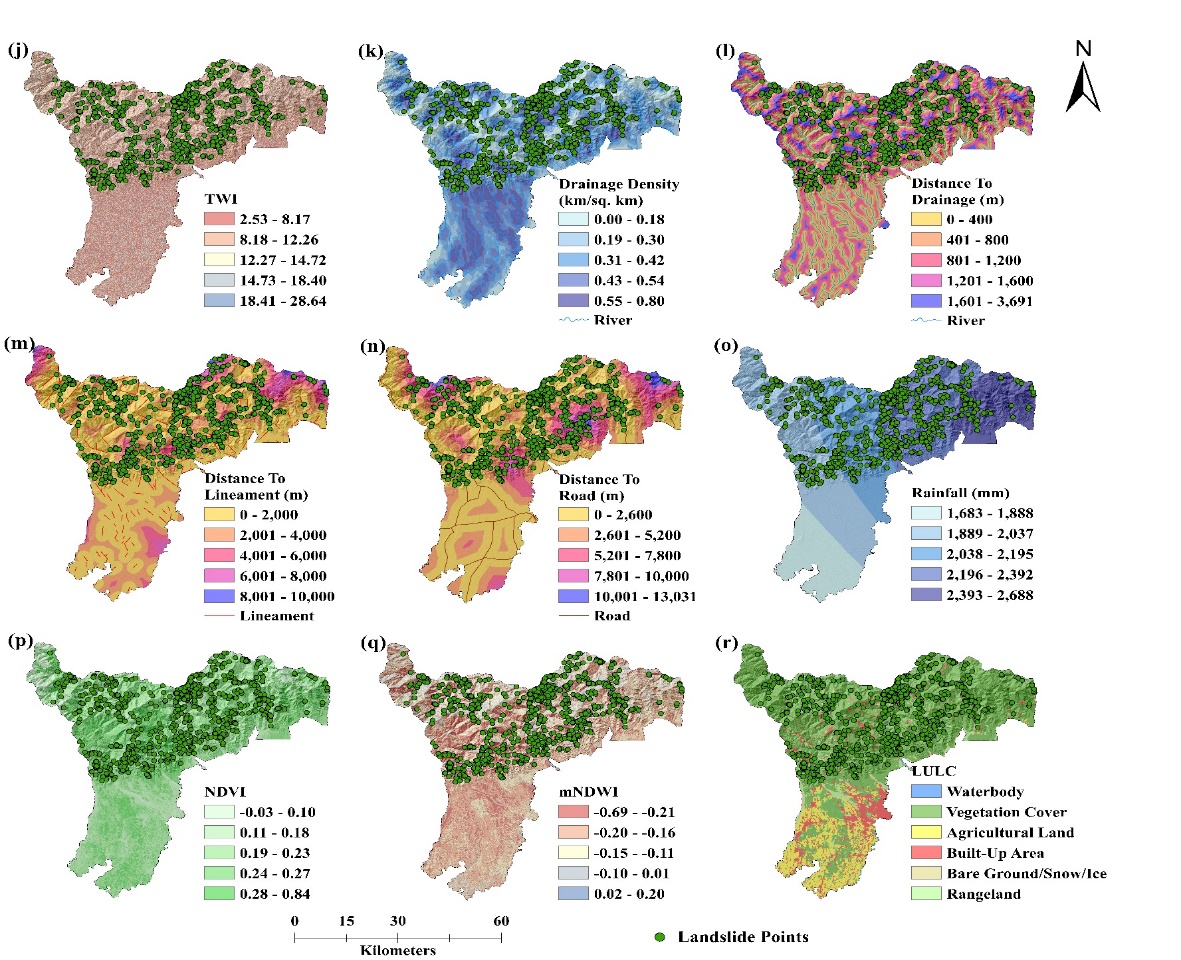


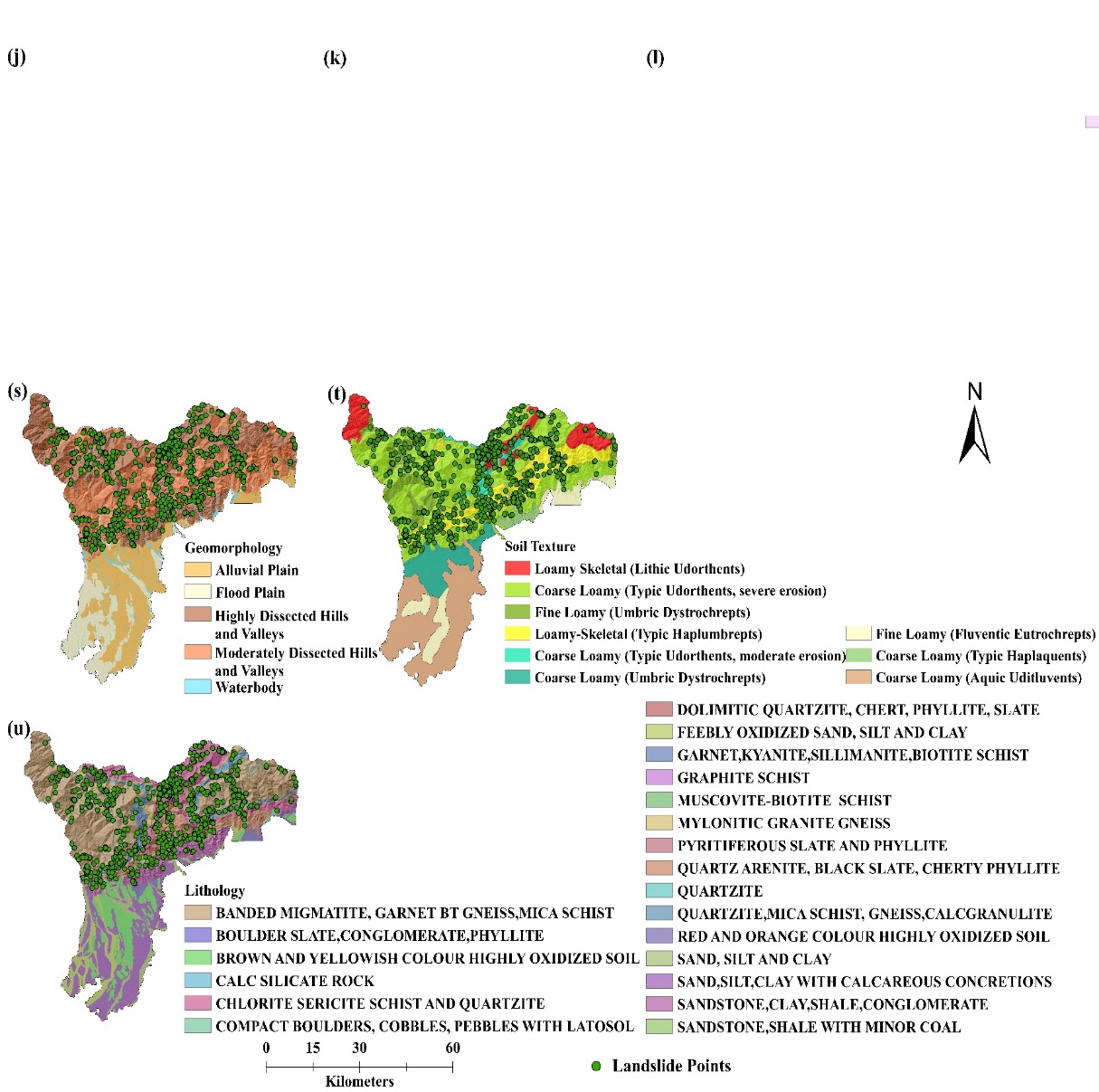


**Figure S1.** Landslide conditioning factors (a) Elevation (Ele), (b) Slope (Sl), (c) Slope Aspect (SA), (d) Profile Curvature (PC), (e) Roughness (Rn), (f) Stream Power Index (SPI), (g) Sediment Transport Index (STI), (h) Topographic Position Index (TPI), (i) Topographic Ruggedness Index (TRI), (j) Topographic Wetness Index (TWI), (k) Drainage Density (DD), (l) Distance to Drainage (DTD), (m) Distance to Lineament (DTL), (n) Distance to Road (DTR), (o) Rainfall (RF), (p) Normalised Difference Vegetation Index (NDVI), (q) Modified Normalised Difference Water Index (mNDWI), (r) Landuse Landcover (LULC), (s) Geomorphology (GM), (t) Soil Texture (ST) and (u) Lithology (LITH).

[Created by ArcGIS 10.4.1; Esri. (2016). ArcGIS Desktop: Release 10.4.1 [Software]. Environmental Systems Research Institute. <https://www.esri.com> ]

**Supplementary S2: Details of the models tested in the current study.**

***Logistic Regression (LR):***

Logistic regression is a widely used statistical model that is primarily known for binary classification problems and focuses on predicting whether an instance falls into one of two different groups. This model assumes that the relationship between the independent factors and the binary outcome may be encapsulated by the logistic (sigmoid) function. It transforms the linear combination of input data into a range of probabilities ranging from 0 to 1. The resulting probability serves as a valuable metric, signifying the possibility of an instance belonging to the positive class [11, 26].

The hyperparameters specified for the LR model are as follows: The optimization algorithm (solver) is set to 'newton-cg'. The penalty is set to 'l2,' implying ridge regularization. The maximum number of iterations for the optimization process is restricted to 100. Class weights being 'None', indicate equal weights for all classes. The inverse of regularization strength, represented by 'C,' is set to 10, suggesting a rather poor regularization. The mathematical formula encoding the logistic regression model is stated in Eqs. (11).

$$P\left( Y=1 \right)=\frac{1}{1+ⅇ^{-\left( \beta_{0}+\beta_{1}X_{1}+\beta_{2}X_{2}+\ldots+B_{n}X_{n} \right)}} (11)$$

Here, $P\left( Y=1 \right)$ is the probability of the dependent variable being class 1 (positive class), $\beta_{0}$ is the intercept, $\beta_{i}$ are the coefficients and $X_{i}$ are the input features.

***Support Vector Machine (SVM):***

SVM is a powerful supervised machine learning algorithm generally used for both classification and regression problems. The main purpose of SVM is to find an optimal hyperplane that best separates the data points belonging to various classes in a high-dimensional feature space. This hyperplane is positioned to maximize the margin, which is the distance between the hyperplane and the nearest data points from each class [57].

SVM with a polynomial kernel is particularly effective when the underlying decision boundary in the original feature space is nonlinear. The cross-validation accuracy of the SVM model reaches its maximum when the gamma parameter is set at 1, which increases the complexity of the decision boundary, with a greater value potentially contributing to overfitting. The regularization parameter (`C`), set as 10, finds a balance between producing a smooth decision border and reliably classifying training points. The polynomial kernel function and the decision function of SVM are defined in Eqs. (12) and (13), respectively.

$$K\left( x,y \right)=\left( x\cdot y+c \right)^{d} (12)$$

Where $x$ and $y$ are the input vectors, $c$ is the bias term, and $d$ is the degree of the polynomial.

$$f\left( x \right)=sign\left( \sum_{i=1}^{n} a_{i}y_{i}K\left( x,x_{i} \right)+b \right) (13)$$

Here, $a_{i}$ and $y_{i}$ are the Lagrange multipliers and class labels for the support vector $x_{i}$, $K\left( x,x_{i} \right)$ is the polynomial kernel function and $b$ is the bias term.

***Random Forest (RF)***

A popular tree-based ensemble learning technique called random forest can handle both regression and classification tasks quite well. To compute the mode of the classes in the case of classification, this approach constructs several decision trees during training [1, 46]. Choosing a random subset of features for every tree and bootstrapping the data, adds unpredictability. This unpredictability ensures that the individual trees are diverse, collectively generating a strong and accurate model.

The selected hyperparameters for the RF model significantly impact the model’s behaviour and performance. The ensemble offers resilience with 100 decision trees. The `min_samples_split` and `min_samples_leaf` parameters, set at 4 and 1, respectively, restrict the size of splits during tree construction, aiding in minimizing overfitting. The `max_samples` parameter, set to 1, indicates that each tree is trained on the full dataset. With `max_features` at 0.6, the method introduces randomness by considering a subset of features at each split. The model permits trees to expand up to maximum depth. The 'gini' criterion measures split quality based on impurity, useful for classification jobs. Enabling bootstrapping offers extra variation in the training subsets. An aggregation over the decision trees is the mathematical expression for the random forest model's decision function. Eqs. (14)

$$F\left( X \right)=mode\left\{ f_{1}\left( X \right)+f_{2}\left( X \right)+\cdots+f_{N}\left( X \right) \right\} (14)$$

Here, the prediction is given by $F\left( X \right)$, $mode$ indicates the class that appears most frequently among the predictions provided by each tree, and $N$ is the total number of trees in the random forest ensemble. Each $f_{i}\left( X \right)$ corresponds to the output of the $i$-th decision tree, which is trained on a random subset of features and a bootstrapped sample of the dataset.

***Extra Trees (ET)***

Extra Trees, short for Extremely Randomized Trees, is an ensemble learning method that belongs to the broader category of decision trees. It is an extension of the Random Forest algorithm, designed to introduce additional randomness during the tree-building process. In Extra Trees, not only are random subsets of features considered for each split but the split points are also chosen at random. This heightened level of randomness enhances the diversity among the individual trees in the ensemble, making Extra Trees robust against overfitting [61, 62].

The selection of the optimal split point in Extra Trees involves evaluating the quality of each potential split using a criterion such as Gini impurity or information gain. The algorithm selects the split point that minimizes the impurity in the resulting child nodes. The randomness in the split point selection adds an extra layer of unpredictability, contributing to the algorithm's overall effectiveness.

The equation for split point selection in Extra Trees is akin to other decision tree algorithms, where a criterion is used to assess the impurity of a node. Let's denote the impurity measure as $I$, and for a given node $t$, it can be expressed as follows:

$$I\left( t \right)=p\left( t \right).I_{G}\left( t \right) (15)$$

where $p\left( t \right)$ is the proportion of samples in node $t$ relative to its parent node, and $I_{G}\left( t \right)$is the impurity measure (e.g., Gini impurity) for node t. The algorithm then iterates through a random subset of features and potential split points to find the combination that minimizes the weighted impurity across all nodes.

In summary, Extra Trees' distinctive feature lies in its introduction of extra randomness during both feature and split point selection, contributing to a more diverse and robust ensemble of decision trees. The hyperparameters employed in the Extra Trees model closely resemble those of the Random Forest model, as outlined in Table 3.

***Gradient Boosting (GB)***

Gradient boosting is a powerful machine learning ensemble technique that combines the strengths of multiple weak learners to create a robust predictive model. The algorithm works by sequentially adding decision trees to the ensemble, with each tree attempting to correct the errors of the previous ones. The concept of "gradient" in gradient boosting refers to the use of gradient descent optimization to minimize the loss function, which measures the difference between the predicted values and the actual values of the target variable [5, 44]. The basic equation for gradient boosting can be expressed as follows:

$$F_{m}\left( x \right)=F_{m-1}\left( x \right)+a\cdot h_{m}\left( x \right) (16)$$

Where, $F_{m}\left( x \right)$ is the ensemble model at iteration *m*, $F_{m-1}\left( x \right)$ is the ensemble model at iteration $m-1$, $a$ is the learning rate, controlling the contribution of each weak learner, $h_{m}\left( x \right)$ is the weak learner (typically a decision tree) added at iteration $m$.

The algorithm iteratively improves the model by fitting new weak learners to the residuals of the previous model. This process continues until a predefined number of iterations is reached or the model achieves satisfactory performance. Gradient boosting is widely used in various applications, including regression and classification tasks, due to its ability to handle complex relationships in data and produce highly accurate predictions. Significant hyperparameters to consider for the Gradient boosting model are ‘n_estimators’ which is set to 150, determining the number of boosting stages, and ‘learning_rate’ which is set at 0.2, controlling the contribution of each tree to the final prediction. The parameters such as ‘min_samples_split’, ‘min_samples_leaf’, ‘max_features’, and ‘max_depth’ exhibit similar functionalities to their counterparts in the previous models. The ‘subsample’ parameter, set at 0.7, determines the proportion of samples that are utilized for training each base learner.

***Extreme Gradient Boosting (XGBoost)***

XGBoost (Chen & Guestrin, 2016) is a machine learning algorithm based on a gradient boosting framework, which is used for both regression and classification problems. This framework uses gradient boosting to create a series of weak learners, usually decision trees, and then gradually improves prediction accuracy [10, 14, 22, 58]. The formulation of XGBoost combines the capabilities of gradient boosting and regularization techniques to generate robust and accurate models Eqs. (17).

$$F\left( X \right)=\sum_{m=1}^{M} f_{m}\left( X \right) (17)$$

Where, $F\left( X \right)$ is the outcome, $M$ is the total number of weak learners, and $f_{m}\left( X \right)$ is the prediction of the $m$-th tree. Each tree is trained to optimize the loss of its predecessors, thereby adapting and boosting the model's overall accuracy.

Because it balances predictive performance and model complexity, XGBoost's efficacy can be attributed to the regularization aspects it incorporates into the objective function. It is decided to formulate the objective function as Eqs. (18):

$$Obj=\sum_{i=1}^{n} L\left( y_{i},\hat{y}_{i} \right)+\sum_{m=1}^{M} \Omega\left( f_{m} \right) (18)$$

Here, $L\left( y_{i},\hat{y}_{i} \right)$ is the loss function, evaluating the difference between the actual label $y_{i}$ and the predicted label $\hat{y}_{i}$, while $\Omega\left( f_{m} \right)$ represents the regularization term for the $m$-th tree.

The XGBoost model employs several hyperparameters to fine-tune its performance. The number of boosting rounds is set to 150. The learning rate at 0.3 regulates the step size during the optimization process. Other hyperparameters like ‘min_child_weight’, ‘gamma’, and ‘colsample_bytree’ regulate things like node splitting conditions and column subsampling.

***Stacked Meta Classifier***

A meta-model is a model that is trained on the predictions of other base models. Instead of making predictions directly based on the input features, a meta-model takes the predictions from many base models as its input and provides a final output. In stacking, for example, many base models are trained on the dataset, and their predictions become the input for a higher-level meta-model. The meta-model learns how to integrate or weigh the predictions from the underlying models to get a more accurate and robust final prediction.

We employed a stacking ensemble strategy, which is an aggregation of all six different machine learning models discussed earlier. The ensemble employs a logistic regression model as the final estimator, effectively combining the capabilities of individual models. A stratified K-fold cross-validation technique with five folds is applied to robustly test the ensemble's performance. During training, predictions from the base models are used as features for the logistic regression meta-classifier, allowing the ensemble to acquire appropriate weights for each model's contribution. This methodology seeks to boost prediction accuracy by capturing diverse patterns existing in the data. The stacking ensemble, with logistic regression as the final layer, adds a higher level of sophistication and adaptability, perhaps leading to improved generalization on unknown data.

| **Table S1.** Feature rankings, selection status of the models, and ensemble vote counts | | | | | | | | | | | | | | | | |
| --- | --- | --- | --- | --- | --- | --- | --- | --- | --- | --- | --- | --- | --- | --- | --- | --- |
| Landslide Influencing Factors | Encoded Categorical Factors | Abbreviation | Logistic Regression | | Support Vector Machine | | Random Forest | | Extra Trees | | Gradient Boosting | | XGBoost | | Vote Counts | Status |
|  |  |  | Selection | Ranking | Selection | Ranking | Selection | Ranking | Selection | Ranking | Selection | Ranking | Selection | Ranking |  |  |
| Elevation | - | - | T | 1 | T | 1 | T | 1 | T | 1 | T | 1 | T | 1 | 6 | **Selected** |
| Slope | - | - | T | 1 | T | 1 | T | 1 | T | 1 | T | 1 | T | 1 | 6 | **Selected** |
| Aspect | - | - | F | 8 | F | 24 | F | 4 | F | 14 | T | 1 | F | 7 | 1 | Removed |
| Profile Curvature | - | - | F | 4 | T | 1 | F | 7 | F | 22 | T | 1 | F | 10 | 2 | Removed |
| Roughness | - | - | F | 6 | F | 17 | F | 9 | F | 15 | F | 7 | F | 8 | 0 | Removed |
| SPI | - | - | F | 18 | F | 30 | F | 8 | F | 26 | F | 6 | F | 12 | 0 | Removed |
| STI | - | - | F | 16 | F | 31 | T | 1 | F | 17 | F | 4 | F | 15 | 1 | Removed |
| TPI | - | - | T | 1 | F | 16 | F | 10 | F | 21 | F | 17 | F | 11 | 1 | Removed |
| TRI | - | - | T | 1 | T | 1 | T | 1 | T | 1 | T | 1 | T | 1 | 6 | **Selected** |
| TWI | - | - | T | 1 | F | 18 | F | 6 | F | 20 | T | 1 | F | 6 | 2 | Removed |
| Rainfall | - | - | T | 1 | F | 26 | T | 1 | T | 1 | T | 1 | T | 1 | 5 | **Selected** |
| Dist to Drainage | - | - | T | 1 | T | 1 | F | 3 | F | 8 | T | 1 | F | 3 | 3 | **Selected** |
| Dist to Lineament | - | - | T | 1 | T | 1 | T | 1 | F | 2 | T | 1 | T | 1 | 5 | **Selected** |
| Dist to Road | - | - | T | 1 | T | 1 | T | 1 | T | 1 | T | 1 | T | 1 | 6 | **Selected** |
| Drainage Density | - | - | F | 5 | F | 13 | T | 1 | T | 1 | T | 1 | F | 2 | 3 | **Selected** |
| NDVI | - | - | F | 10 | F | 29 | T | 1 | F | 4 | T | 1 | T | 1 | 3 | **Selected** |
| MNDWI | - | - | T | 1 | F | 7 | T | 1 | F | 9 | T | 1 | F | 5 | 3 | **Selected** |
| Geomorphology | Alluvial Plain | G-AP | T | 1 | T | 1 | F | 2 | T | 1 | F | 9 | F | 33 | 3 | **Selected** |
|  | Flood Plain | G-FP | T | 1 | F | 12 | F | 20 | F | 5 | F | 19 | F | 35 | 1 | Removed |
|  | Highly Dissected Hills and Valleys | G-HDHV | T | 1 | T | 1 | F | 17 | F | 6 | F | 3 | F | 27 | 2 | Removed |
|  | Mass Wasting Products | G-MWP | T | 1 | T | 1 | F | 33 | F | 34 | F | 32 | F | 28 | 2 | Removed |
|  | Moderately Dissected Hills and Valleys | G-MDHV | T | 1 | T | 1 | T | 1 | T | 1 | F | 2 | T | 1 | 5 | **Selected** |
|  | Waterbody River | G-WR | T | 1 | F | 11 | F | 29 | F | 35 | F | 36 | F | 37 | 1 | Removed |
| Lithology | BANDED MIGMATITE GARNET BT GNEISS MICA SCHIST | L-BMGG | F | 15 | F | 21 | F | 13 | F | 11 | F | 27 | F | 4 | 0 | Removed |
|  | BROWN AND YELLOWISH COLOUR HIGHLY OXIDIZED SOIL | L-BYHOS | T | 1 | T | 1 | F | 19 | F | 18 | F | 15 | F | 23 | 2 | Removed |
|  | CALC SILICATE ROCK | L-CSR | F | 11 | F | 34 | F | 45 | F | 46 | F | 33 | F | 29 | 0 | Removed |
|  | CHLORITE SERICITE SCHIST AND QUARTZITE | L-CSSQ | T | 1 | F | 20 | F | 11 | F | 3 | T | 1 | T | 1 | 3 | **Selected** |
|  | COMPACT BOULDERS COBBLES PEBBLES WITH LATOSOL | L-CBCPL | T | 1 | F | 22 | F | 37 | F | 42 | F | 11 | F | 39 | 1 | Removed |
|  | FEEBLY OXIDIZED SAND SILT AND CLAY | L-FOSSC | T | 1 | T | 1 | F | 35 | F | 29 | F | 40 | F | 40 | 2 | Removed |
|  | GARNET KYANITE SILLIMANITE BIOTITE SCHIST | L-GKSBS | T | 1 | F | 9 | F | 39 | F | 39 | F | 20 | F | 38 | 1 | Removed |
|  | MUSCOVITE-BIOTITE SCHIST | L-MBS | T | 1 | F | 10 | F | 41 | F | 41 | F | 25 | F | 36 | 1 | Removed |
|  | MYLONITIC GRANITE GNEISS | L-MGG | F | 14 | F | 19 | F | 32 | F | 37 | F | 8 | F | 34 | 0 | Removed |
|  | PYRITIFEROUS SLATE AND PHYLLITE | L-PSP | F | 19 | F | 35 | F | 42 | F | 48 | F | 18 | F | 32 | 0 | Removed |
|  | QUARTZ ARENITE BLACK SLATE CHERTY PHYLLITE | L-QABSCP | F | 17 | F | 5 | F | 24 | F | 24 | F | 21 | F | 13 | 0 | Removed |
|  | QUARTZITE | L-Q | T | 1 | F | 23 | F | 44 | F | 44 | F | 16 | F | 18 | 1 | Removed |
|  | QUARTZITE MICA SCHIST GNEISS CALCGRANULITE | L-QMSGC | T | 1 | F | 14 | F | 28 | F | 31 | F | 10 | F | 26 | 1 | Removed |
|  | RED AND ORANGE COLOUR HIGHLY OXIDIZED SOIL | L-ROHOS | F | 7 | F | 25 | F | 36 | F | 38 | F | 31 | F | 16 | 0 | Removed |
|  | SAND SILT AND CLAY | L-SSC | T | 1 | T | 1 | F | 38 | F | 30 | F | 29 | F | 31 | 2 | Removed |
|  | SAND SILT CLAY WITH CALCAREOUS CONCRETIONS | L-SSCC | T | 1 | T | 1 | F | 15 | F | 10 | F | 5 | T | 1 | 3 | **Selected** |
|  | SANDSTONE CLAY SHALE CONGLOMERATE | L-SCSC | T | 1 | T | 1 | F | 23 | F | 33 | T | 1 | T | 1 | 4 | **Selected** |
|  | SANDSTONE SHALE WITH MINOR COAL | L-SSMC | F | 2 | F | 36 | F | 43 | F | 45 | F | 23 | F | 22 | 0 | Removed |
| LULC | Waterbody | - | F | 3 | T | 1 | F | 34 | F | 43 | F | 24 | T | 1 | 2 | Removed |
|  | Vegetation Cover | - | T | 1 | F | 8 | F | 21 | F | 19 | F | 14 | T | 1 | 2 | Removed |
|  | Agricultural Land | - | T | 1 | F | 4 | F | 30 | F | 13 | F | 26 | F | 30 | 1 | Removed |
|  | Built-Up Area | - | T | 1 | F | 32 | F | 25 | F | 32 | F | 35 | F | 24 | 1 | Removed |
|  | Bare Ground | - | T | 1 | F | 27 | F | 40 | F | 40 | F | 38 | F | 25 | 1 | Removed |
|  | Snow Ice | - | F | 12 | F | 28 | F | 46 | F | 47 | F | 34 | F | 19 | 0 | Removed |
|  | Rangeland | - | F | 13 | F | 15 | F | 31 | F | 36 | F | 39 | F | 17 | 0 | Removed |
| Soil Texture | Fine Loamy (Fluventic Eutrochrepts) | S-FLE | T | 1 | T | 1 | F | 14 | F | 12 | T | 1 | T | 1 | 4 | **Selected** |
|  | Loamy-Skeletal (Typic Haplumbrepts) | S-LSH | T | 1 | T | 1 | F | 16 | F | 16 | T | 1 | T | 1 | 4 | **Selected** |
|  | Loamy Skeletal (Lithic Udorthents) | S-LSU | F | 9 | F | 6 | F | 12 | F | 7 | F | 30 | T | 1 | 1 | Removed |
|  | Fine Loamy (Umbric Dystrochrepts) | S-FLD | T | 1 | T | 1 | F | 18 | F | 25 | T | 1 | T | 1 | 4 | **Selected** |
|  | Coarse Loamy (Umbric Dystrochrepts) | S-CLD | T | 1 | F | 33 | F | 27 | F | 23 | F | 13 | F | 9 | 1 | Removed |
|  | Coarse Loamy (Typic Haplaquents) | S-CLH | T | 1 | T | 1 | F | 22 | F | 28 | F | 12 | F | 20 | 2 | Removed |
|  | Coarse Loamy (Aquic Uditluvents) | S-CLU | T | 1 | T | 1 | T | 1 | T | 1 | F | 28 | F | 21 | 4 | **Selected** |
|  | Coarse Loamy (Typic Udorthents, moderate erosion) | S-CLU-ME | T | 1 | F | 2 | F | 26 | F | 27 | F | 22 | F | 14 | 1 | Removed |
|  | Coarse Loamy (Typic Udorthents, severe erosion) | S-CLU-SE | T | 1 | F | 3 | F | 5 | T | 1 | F | 37 | T | 1 | 3 | **Selected** |
